# Analysis of the genetic architecture of maize kernel size traits by combined linkage and association mapping

**DOI:** 10.1111/pbi.13188

**Published:** 2019-06-26

**Authors:** Min Liu, Xiaolong Tan, Yan Yang, Peng Liu, Xiaoxiang Zhang, Yinchao Zhang, Lei Wang, Yu Hu, Langlang Ma, Zhaoling Li, Yanling Zhang, Chaoying Zou, Haijian Lin, Shibin Gao, Michael Lee, Thomas Lübberstedt, Guangtang Pan, Yaou Shen

**Affiliations:** ^1^ Key Laboratory of Biology and Genetic Improvement of Maize in Southwest Region Maize Research Institute Sichuan Agricultural University Chengdu China; ^2^ Department of Agronomy Iowa State University Ames IA USA; ^3^ State Key Laboratory of Crop Gene Exploration and Utilization in Southwest China (In preparation) Chengdu China

**Keywords:** maize, kernel size, genome‐wide association study, QTL mapping, co‐localization, functional genes

## Abstract

Kernel size‐related traits are the most direct traits correlating with grain yield. The genetic basis of three kernel traits of maize, kernel length (KL), kernel width (KW) and kernel thickness (KT), was investigated in an association panel and a biparental population. A total of 21 single nucleotide polymorphisms (SNPs) were detected to be most significantly (*P* < 2.25 × 10^−6^) associated with these three traits in the association panel under four environments. Furthermore, 50 quantitative trait loci (QTL) controlling these traits were detected in seven environments in the intermated B73 × Mo17 (IBM) Syn10 doubled haploid (DH) population, of which eight were repetitively identified in at least three environments. Combining the two mapping populations revealed that 56 SNPs (*P* < 1 × 10^−3^) fell within 18 of the QTL confidence intervals. According to the top significant SNPs, stable‐effect SNPs and the co‐localized SNPs by association analysis and linkage mapping, a total of 73 candidate genes were identified, regulating seed development. Additionally, seven miRNAs were found to situate within the linkage disequilibrium (LD) regions of the co‐localized SNPs, of which zma‐miR164e was demonstrated to cleave the mRNAs of *Arabidopsis CUC1*, *CUC2* and *NAC6 in vitro*. Overexpression of zma‐miR164e resulted in the down‐regulation of these genes above and the failure of seed formation in *Arabidopsis* pods, with the increased branch number. These findings provide insights into the mechanism of seed development and the improvement of molecular marker‐assisted selection (MAS) for high‐yield breeding in maize.

## Introduction

Maize (*Zea mays*) is one of the most important staple crops, which serves as a resource for human nutrition, animal feed and bioenergy (Godfray *et al*., 2010). Therefore, improving maize yield is an essential measure to ensure food security, considering the global decrease in cultivated areas. Kernel size, a major component determining kernel weight, is one of the most important yield traits, which includes kernel length (KL), kernel width (KW) and kernel thickness (KT; Li *et al*., 2013a; Liu *et al*., 2016c). Compared with grain yield itself, kernel size traits show higher heritability and better stability across environments (Messmer *et al*., 2009; Raihan *et al*., 2016). Accordingly, elucidation of the genetic basis of kernel traits will facilitate in the elucidation of the regulatory mechanisms involved in maize seed development and the design of strategies to improve corn yield.

Currently, quantitative trait loci (QTL) mapping and genome‐wide association studies (GWAS) are effective tools for analysing the genetic structure of complex quantitative traits. In the last few decades, many loci or candidate genes associated with kernel‐related traits have been identified by linkage and/or association mapping (Cook *et al*., 2012; Yang *et al*., 2016). For instance, Liu *et al*. (2017a) identified 729 QTL for kernel size and weight in 10 recombinant inbred line (RIL) populations (Liu *et al*., 2017a). Li *et al*. (2018a) identified 27 associated loci involving 39 candidate genes for amylose content using an association population (Li *et al*., 2018a). Li *et al*. (2018b) identified 74 loci significantly associated with kernel oil concentration and fatty acid composition by GWAS (Li *et al*., 2013b). Zhang *et al*. (2017a) detected 108 QTL for eight ear and grain traits by combined linkage and association mapping (Zhang *et al*., 2017a). However, these researches in maize are relatively fewer compared to those involving rice. Numerous genes involved in rice kernel traits have been isolated and functionally characterized using map‐based cloning and GWAS strategies, such as *LONG KERNEL 3* (*GS3*), *GRAIN SIZE 5* (*GS5*), *GRAIN WEIGHT 2* (*GW2*), *GRAIN WIDTH 5* (*GW5*), Grain Width/Length QTL on chromosome 7 (*GW7*/*GL7*) and *GRAIN WIDTH 8* (*GW8*; Fan *et al*., 2006; Li *et al*., 2011; Mao *et al*., 2010; Si *et al*., 2015; Song *et al*., 2007; Wang *et al*., 2012b, 2015a,2015b; Weng *et al*., 2008). However, in maize, most of the functional genes involved in kernel development were identified using maize mutants, such as *opaque2(o2)*, *defective kernel 1(dek1), empty pericarp2 (emp2), empty pericarp4(emp4), empty pericarp5(emp5), empty pericarp16 (emp16), shrunken1(sh1), glutamine synthetase1(gln1), embryo defective14* and *U6 biogenesis‐like1* (Echt and Chourey, 1985; Fu *et al*., 2002; Gutiérrez‐Marcos *et al*., 2007; Li *et al*., 2015, 2017; Lid *et al*., 2002a,2002b; Liu *et al*., 2013; Maitz *et al*., 2000; Martin *et al*., 2006; Thévenot *et al*., 2005; Xiu *et al*., 2016; Zhou *et al*., 2016). Moreover, some functional genes that control maize kernel weight and size were identified using homology‐based cloning, including the orthologues of rice *GW2* and *GS3*, *ZmGW2* and *ZmGS3* (Li *et al*., 2010a,2010b). However, these mutant‐ or homology‐based cloned yield‐related genes are greatly limited in application to improving maize yield by molecular marker‐assisted (MAS) breeding due to the lack of identified superior allelic variations. To date, only a few genes that influence maize kernel traits have been isolated through positional cloning, such as *ZmCKX10*, which was cloned by fine mapping of a major QTL (qKL1.07) for kernel length (Qin *et al*., 2016). Therefore, illustrating the genetic architecture of maize yield and revealing superior alleles by combination of GWAS and QTL mapping will contribute to MAS and genomic selection (GS) breeding of high‐yield maize as well as to identify functional genes that control maize yield.

The development of maize kernels is regulated by a large number of genes at the transcriptional and post‐transcriptional levels (Chen *et al*., 2014; Li *et al*., 2016). Plant microRNAs (miRNAs) are endogenous ~22‐nt RNAs that play important regulatory roles at the post‐transcriptional level during development and stress response (Chen, 2009). The function of miRNAs is to bind its target genes and cleave their mRNAs or inhibit their translation (Park *et al*., 2002). Currently, miRNAs have attracted much attention for their importance in various development processes. For example, a dynamic expression profile of miRNAs was found to occur during maize kernel development (Li *et al*., 2016). Liu *et al*. (2014a) combined small RNA and degradome sequencing identified miRNAs and their target genes in developing maize ears, confirming 22 conserved miRNA families and discovering 26 novel miRNAs that regulate ear development (Liu *et al*., 2014a). Moreover, the overexpression of miR156 in switchgrass was found to improve biomass production (Fu *et al*., 2012). The miR157/*SPL* axis has been proven to control floral organ growth and ovule production by regulating MADS‐box genes and auxin signal transduction to improve cotton yield (Liu *et al*., 2017b). Zhu *et al*. (2009) revealed that miR172 causes loss of spikelet determinacy, floral organ abnormalities and seed weight reduction in rice (Zhu *et al*., 2009). Plant miRNAs have become important regulatory factors of plant genes, which have the potential to improve complex traits such as crop yield. However, the identification of miRNA loci associated with target traits by GWAS and QTL has not been reported to date. In this study, candidate miRNAs associated with kernel size traits were excavated according to the co‐localized region of GWAS loci and QTL. The findings of this study will improve our understanding of the molecular mechanism underlying kernel yield formation in maize.

In the present study, we used an association panel, including 310 maize inbred lines and an intermated B73 × Mo17 (IBM) Syn10 doubled haploid (DH) population containing 265 DH lines to: (i) identify genetic loci and candidate genes for KL, KT and KW in multiple environments by GWAS; (ii) detect the QTL for KL, KT and KW traits in different environments using an ultra‐high‐density bin map; and (iii) determine co‐localized candidate genes associated kernel size by joint linkage mapping and GWAS. Remarkably, seven miRNAs were found to situate within the linkage disequilibrium (LD) regions of the co‐localized SNPs, of which zma‐miR164e was demonstrated to cleave the mRNAs of *Arabidopsis CUC1*, *CUC2* and *NAC6 in vitro*. Overexpression of zma‐miR164e resulted in the down‐regulation of these genes above and the failure of seed formation in *Arabidopsis* pods, with the increased branch numbers. The present study aims to improve our understanding of the genetic architecture and molecular mechanism of maize kernel yield and contribute to the improvement for kernel yield in maize.

## Results

### Phenotype description for kernel size traits in the association panel and linkage population

**Figure 1:**
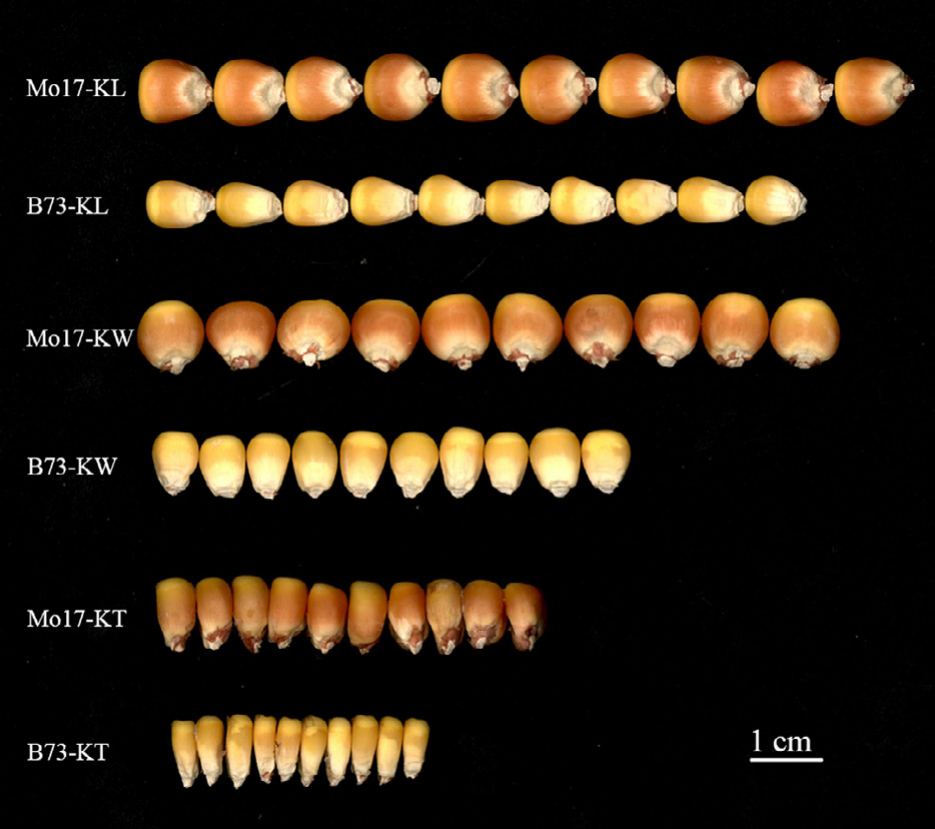
Phenotypes of kernel size traits and variations of kernel size between two parental lines of the IBM Syn 10 DH population. Phenotypes of KL, KW and KT illustrated with 10 kernels of the two parental lines in IBM Syn 10 DH population. Bar = 1 cm.

Generally, abundant variations in kernel size traits were observed in the association panel and the biparental population (Tables S1, S2; Figure 1). KL, KW and KT ranged from 6.50 to 13.60 cm, 4.81 to 9.93 cm and 15.91 to 33.29 mm, with a mean of 9.65, 7.27 cm and 23.21 mm, respectively, across different environments in the association panel (Table S1). For the IBM population, KL, KW and KT had a range from 7.12 cm to 13.07 cm, 4.82 cm to 10.45 cm and 3.43 cm to 4.99 cm, with an average of 10.50 cm, 7.15 cm and 4.42 cm, respectively, across various environments. The broad‐sense heritability (*H* ^2^) of the three‐grain traits ranged from 81.61 (%) to 88.08 (%) in the association panel, and 73.03 (%) for KL, 84.06 (%) for KW and 93.61 (%) for KT in the IBM population. Skewness and kurtosis indicated that these phenotypes all conformed to a normal distribution in the two populations. In the association panel, KW was consistently significantly positively correlated with KT [*r* = 0.293 (E1a), 0.217 (E2a), 0.309 (E3a); *P* < 0.01] across the three environments, and KL was significantly negatively correlated with KT [*r* = −0.252 (E2a), −0.127 (E3a); *P* < 0.05] across two of the environments (Table S3). In the IBM population, KL was consistently significantly positively correlated with KW at the level of *P* < 0.05, and the correlation coefficient was 0.158–0.594 across the six environments. Moreover, KW was consistently significantly positively correlated with KT [*r* = 0.186 (E4a), 0.196 (E5a), 0.136 (E6a); *P* < 0.05] for all three of the environments in the IBM population (Table S4). These results suggested that KL, KW and KT were coordinately developed to regulate kernel size and weight in maize. For each of the traits, there was a highly significantly positive correlation of the phenotypic values between each of the two environments in both populations (Tables S5 and S6). It indicated that the investigated phenotypes were reliable for the genetic architecture dissection of kernel size traits in maize.

### Genetic loci and candidate genes identified in the association panel

**Figure 2:**
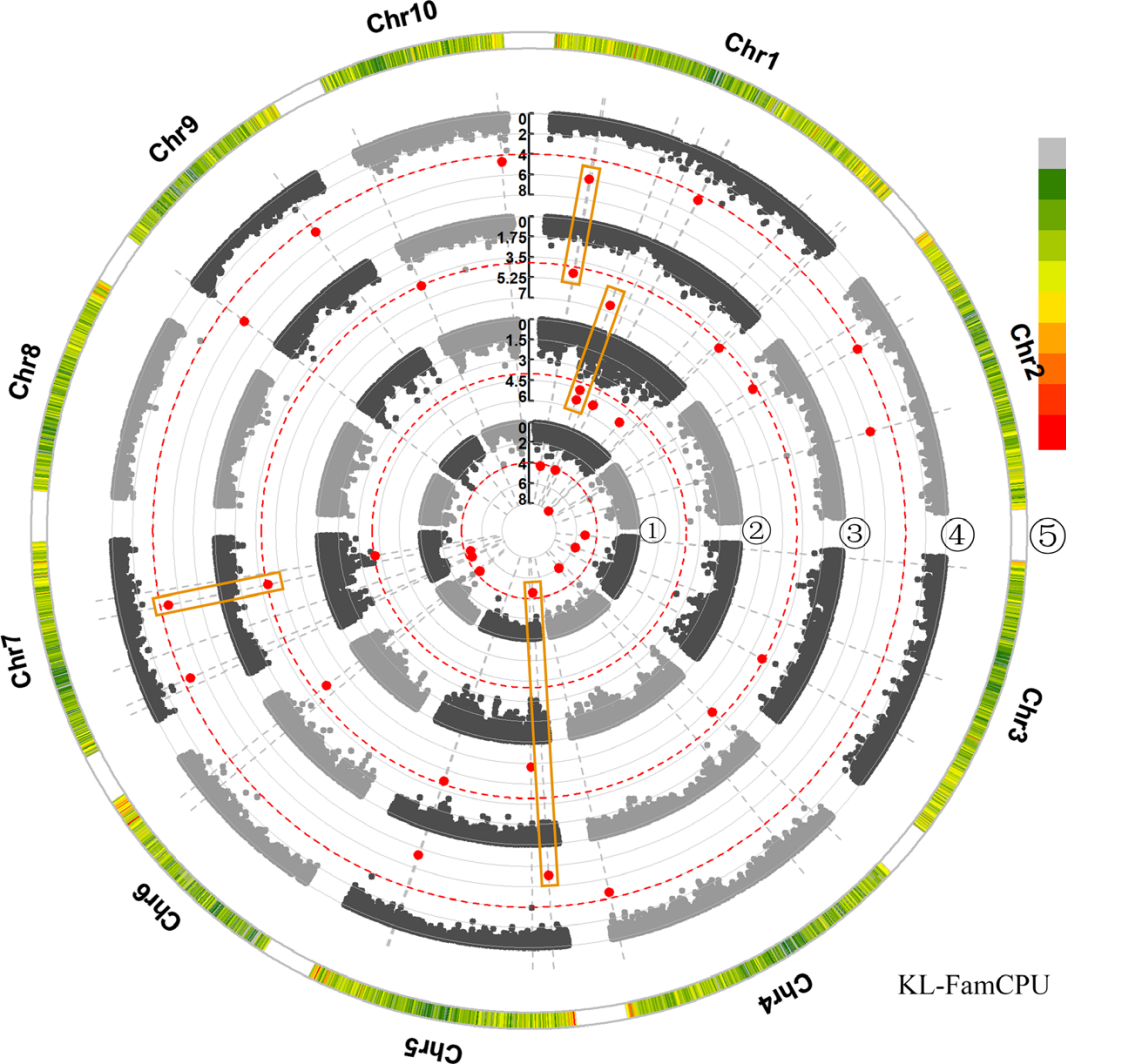
Manhattan plots of the association analysis for KL in four environments by FarmCPU. Manhattan plot of KL on 10 chromosomes for the association analysis across four environments by FarmCPU. The dotted red line indicates the significance threshold of *P*‐value 1 × 10^−4^. The significant SNPs are labelled with red dots. ⑤, distribution of SNP markers on 10 chromosomes in association pool, the colour represents the density of the SNP markers; ①–④, represent different environments: ①, 2016 Jinghong; ②, 2016 Hongya; ③, 2016 Ya'an; ④, BLUP. Stable‐effect SNPs co‐detected in a multi‐environment are shown in orange rectangle‐shaped boxes.

The results of analysis using STRUCTURE revealed that the 310 maize inbred lines could be divided into four subpopulations, which agrees with the findings of a previous study (Zhang *et al*., 2016). For the association panel, LD decay reached 220 kb (*r* ^2^ = 0.2), which is also consistent with results of the previous study (Zhang *et al*., 2016). The GWAS used 39,354 SNPs to perform the elucidation of the genetic architecture of kernel size traits. We used three association analysis methods to balance false positives and false negatives in the present study. To explore the kernel size‐related SNPs with the top significances, we set the threshold as 2.25 × 10^−6^ based on the effective number of SNPs and familywise error rate as α = 0.05. A total of 21 SNPs significantly associated with the three traits were identified by three association analysis methods, of which two SNPs were detected by GAPIT, one by TASSEL and 20 by FarmCPU (Table 1, and Figures 2; Figures S1 and S2). Two SNP markers on chromosome 1, PZE‐101129119 and PZE‐101108339, were co‐detected by two models. Specifically, PZE‐101129119 was detected by GAPIT (E4a, *P* = 2.15 × 10^−6^) and TASSELL (E1a, *P* = 1.26 × 10^−6^), which was significantly associated with KL, whereas PZE‐101108339 was co‐identified by GAPIT (E3a, *P* = 8.64 × 10^−7^) and FarmCPU (E3a, *P* = 3.74 × 10^−7^), which was significantly related to KL. However, 19 SNPs were specifically detected by the FarmCPU model, which were associated with KL, KW and KT (Table 1). Then, we used these 21 unique SNPs to identify candidate genes, of which four SNPs were located in the intergenic regions, and 17 were situated in intragenic regions. The 17 candidate genes involved nine for KL, one for KW and seven for KT (Table 2), including the orthologs/homologs of five genes controlling kernel development as EMBRYO DEFECTIVE 2733 (Shen *et al*., 2013), pentatricopeptide repeat‐containing protein (Yang *et al*., 2017), E3 ubiquitin‐protein ligase HRD1A (Song *et al*., 2007), ubiquitin‐activating enzyme E12 (Du *et al*., 2014; Salceda and Caro, 1997) and CLAVATA3/ESR‐related protein 25 (Fiume and Fletcher, 2012).

**Table 1:** Top‐significance SNPs associated with maize kernel size traits detected by GWAS using three models (GAPIT, TASSEL and FarmCPU)

Num	SNP ID	SNP_position_v2(bp)	Chr	Trait	*P*‐value	PVE	Env.	Model
1	PZE‐101129119	164640160	1	KL	2.15E−06	0.3099	E4a	GAPIT
					1.26E−06	0.0944	E1a	TASSEL
2	PZE‐101108339	115012268	1	KL	8.64E−07	0.2768	E3a	GAPIT
					3.74E−07	0.0001	E3a	FarmCPU
3	SYN26797	18426957	7	KT	3.77E−09	0.0151	E1a	FarmCPU
4	SYN434	289546922	1	KL	1.15E−08	0.0039	E1a	FarmCPU
5	PZE‐110055291	106051168	10	KT	1.47E−08	0.0166	E1a	FarmCPU
6	SYN28965	160819237	1	KT	2.33E−08	0.0166	E1a	FarmCPU
7	PUT‐163a‐29945603‐1815	167062100	5	KL	4.65E−08	0.0241	E4a	FarmCPU
8	SYN1412	15323770	5	KL	8.17E−08	0.0151	E4a	FarmCPU
9	PZE‐110034247	65089069	10	KT	9.78E−08	0.0002	E1a	FarmCPU
10	PZE‐105045953	33978605	5	KL	2.02E−07	0.0008	E3a	FarmCPU
11	SYN32618	120118588	10	KT	2.17E−07	0.0047	E1a	FarmCPU
12	PZE‐107083070	137701592	7	KT	2.74E−07	0.0250	E1a	FarmCPU
13	SYN19035	50343804	5	KW	4.27E−07	0.0024	E4a	FarmCPU
14	PZE‐109000187	479709	9	KL	5.54E−07	0.0027	E4a	FarmCPU
15	PZE‐104143542	232139827	4	KT	5.80E−07	0.0043	E1a	FarmCPU
16	SYN11831	143268372	2	KL	7.66E−07	0.0016	E4a	FarmCPU
17	PZE‐104121926	198929997	4	KW	9.72E−07	0.0158	E4a	FarmCPU
18	SYN3006	45780048	1	KL	1.05E−06	0.0151	E4a	FarmCPU
19	SYN33300	54509254	4	KL	1.11E−06	0.0008	E1a	FarmCPU
20	PZE‐103059745	111867963	3	KL	1.52E−06	0.0009	E1a	FarmCPU
21	PUT‐163a‐71311544‐3115	172351409	8	KT	1.94E−06	0.0181	E1a	FarmCPU

Num, number; Chr, chromosome; Env., environment: E1a is 2016 Jinghong; E2a is 2016 Hongya; E3a is 2016 Ya'an; E4a is BLUP; PVE, phenotypic variation explained.

**Table 2:** Candidate genes associated with the top significant SNPs involved in maize kernel size traits

Num	Candidate gene_v4	Biotype	Gene_start	Gene_end	Strand	SNP	Position_v4 (bp)	Chr	Trait	Annotation
1	Zm00001d030895	IN	166287332	166290184	−	PZE‐101129119	166289395	1	KL	Adenine phosphoribosyltransferase 1 chloroplastic
2	Zm00001d034507	IN	294961663	294963739	+	SYN434	294962348	1	KL	CLAVATA3/ESR‐related protein25/ZmCLE4A
3	Zm00001d016640	IN	170975264	170982646	−	PUT‐163a‐29945603‐1815	170975750	5	KL	TUDOR‐SN protein 1
4	Zm00001d013631	IN	15866934	15871877	−	SYN1412	15867026	5	KL	TATA‐box‐binding protein 2
5	Zm00001d014180	IN	35160223	35174053	−	PZE‐105045953	35160866	5	KL	Flowering locus K homology domain
6	Zm00001d004898	IN	147703036	147707778	−	SYN11831	147703325	2	KL	E3 ubiquitin‐protein ligase HRD1A
7	Zm00001d028771	IN	46017582	46024037	−	SYN3006	46019273	1	KL	Ubiquitin‐activating enzyme E1 2
8	Zm00001d049979	IN	56765844	56767670	+	SYN33300	56766552	4	KL	EMBRYO DEFECTIVE 2733/EMB2733
9	Zm00001d041108	3′‐UTR	97773979	97798160	+	PZE‐103059745	97799302	3	KL	Probable Ufm1‐specific protease
10	Zm00001d014530	IN	51914095	51915783	+	SYN19035	51915123	5	KW	Phenolic glucoside malonyltransferase 1
11	Zm00001d019145	IN	19093571	19096947	−	SYN26797	19095064	7	KT	Leucine‐rich repeat receptor‐like tyrosine‐protein kinase PXC3
12	Zm00001d025152	IN	106764011	106766200	−	PZE‐110055291	106766132	10	KT	Pentatricopeptide repeat‐containing protein/PPR
13	Zm00001d030833	3′‐UTR	162240102	162241109	+	SYN28965	162241249	1	KT	Transcriptional regulator of RNA polII SAGA subunit
14	Zm00001d025522	IN	121168118	121172140	+	SYN32618	121171324	10	KT	Putative FKBP‐type peptidyl‐prolyl cis‐trans isomerase family protein
15	Zm00001d021010	5′‐UTR	140312368	140312751	+	PZE‐107083070	140312336	7	KT	Vegetative cell wall protein gp1
16	Zm00001d053628	IN	237227892	237245986	−	PZE‐104143542	237228035	4	KT	Ribosomal RNA‐processing protein 8
17	Zm00001d012640	IN	177653401	177663257	+	PUT‐163a‐71311544‐3115	177659431	8	KT	Jacalin‐related lectin 3

Chr, chromosome; Num, number.

To discover the stable SNPs co‐detected in a multi‐environment, we then lowered the threshold of *P*‐value to 1.0 × 10^−4^, according to the previous report (Liu *et al*., 2016a). A total of 13 SNPs were significantly correlated with these kernel size traits across multiple environments (Figures 2; Table S7, Figures S1 and S2). Among these, two KL‐associated SNPs (PZE‐101129119 and PZE‐101129122) were repetitively detected in all of the environments, which were co‐identified by all the models. However, SYN18170 (associated with KW) and PZE‐101108339 (associated with KL) were both detected in three environments (E1a, E2a and E4a; E2a, E3a and E4a), which were found by each of the models. Then, we identified nine candidate genes that harboured these 13 SNPs, which included five for KL, three for KW and one for KT (Table S8). Of these, the orthologs/homologs of the candidate genes ubiquitin receptor RAD23c (Peng *et al*., 2015) and ubiquitin‐activating enzyme E12 (Du *et al*., 2014; Salceda and Caro, 1997) have been previously shown to regulate kernel development.

### QTL detected by linkage population

**Figure 3:**
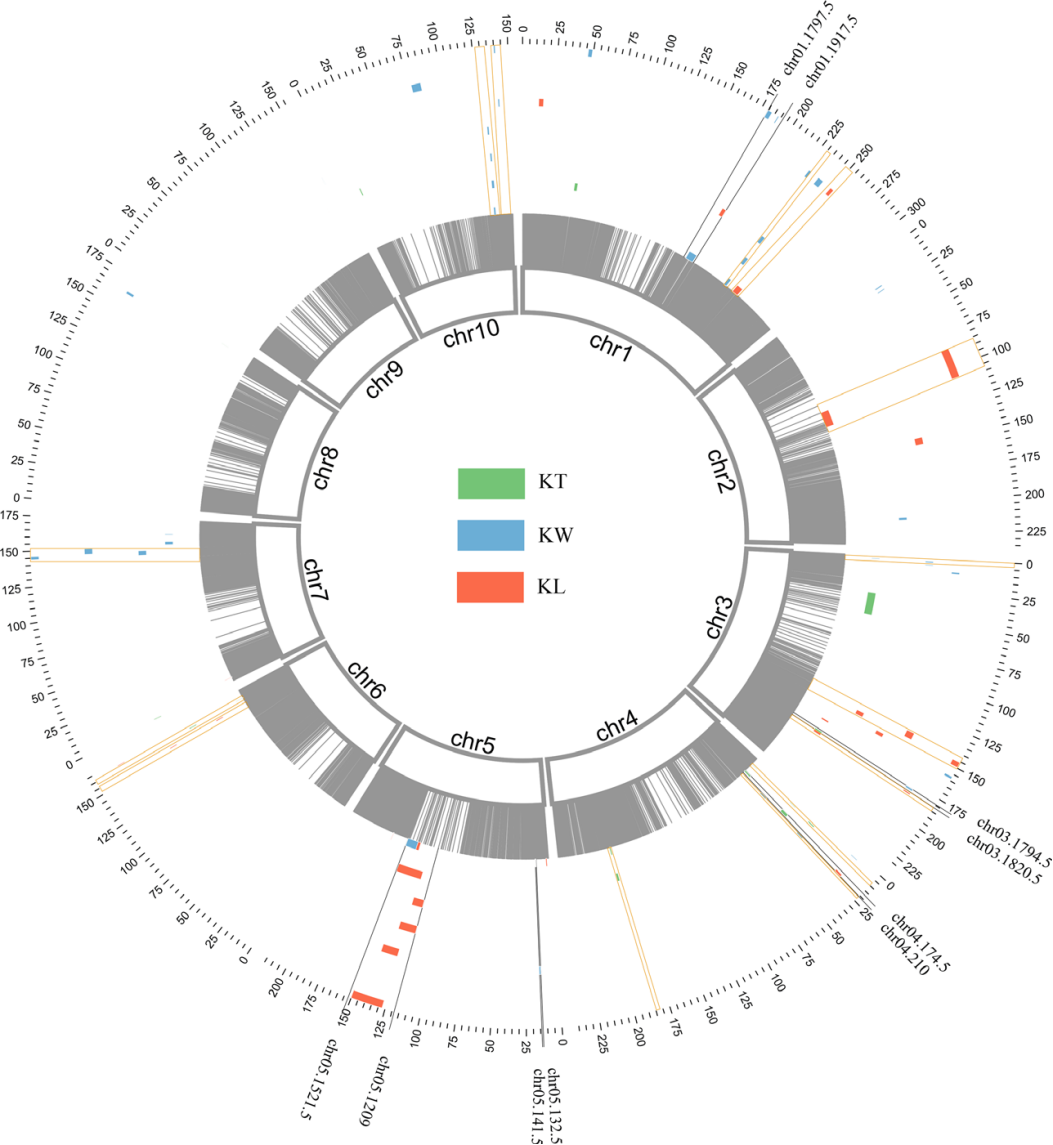
QTL on 10 chromosomes for three kernel size traits across seven environments. Circos graph displaying integrated QTL on 10 chromosomes for kernel size traits across seven environments. The innermost ring black strip represents bin markers on 10 chromosomes of maize; the outermost ring scale indicates the physical location on the 10 chromosomes. Seven circles from the outside to the inside represent seven environments, E1b–E7b, respectively. For each environment, different colours represent different traits: green, KL; blue, KW; red, KT. The fan‐shaped region between the two markers (highlighted with the black line) represents each pleiotropic QTL. The stable QTL co‐detected in a multi‐environment are shown in orange rectangle‐shaped boxes.

Based on single‐environment QTL analysis, a total of 50 QTL distributed across 10 maize chromosomes were identified for the three traits, including 15 QTL for KL, 21 QTL for KW, nine QTL for KT and five QTL for multiple traits (Figure 3; Table S9). The confidence intervals of these QTL spanned physical distances from 0.20 to 24.80 Mb, with an average of 3.82 Mb, by referring to the B73 RefGen_v2 genome. The proportion of phenotypic variations explained by these individual QTL ranged from 3.48% to 10.11% for KL, from 3.44% to 8.43% for KW and from 3.38% to 15.04% for KT (Table S9). To analyse the overlaps between different QTL identified in each of the environments, we compared the confidence intervals of the mapped QTL. When two QTL overlapped, these were considered to be a single unique QTL. A total of 19 overlapping QTL were detected across different environments or across different traits in this study. Furthermore, there were 18 QTL were detected in at least two environments, including six, eight and four QTL for KL, KW and KT, respectively (Table S9). Among these environment‐stable QTL, qKS5‐2 was detected in six of the seven environments, and qKW10‐2 was identified in four environments, whereas qKS1‐1, qKS4‐1, qKL3‐2, qKW7‐1 and qKT4‐2 were detected in three environments. The two QTL, qKL3‐5 (*R* ^2^ = 10.11%) and qKT4‐2 (*R* ^2^ = 15.04%), were identified as major QTL (*R* ^2^ > 10%), which controlled KL and KT, respectively (Table S9). When a QTL is mapped to multiple traits, it was a called pleiotropic QTL (Liu *et al*., 2016c). In this study, five pleiotropic QTL were identified, including qKS1‐1, qKS5‐1 and qKS5‐2 for KL and KW, qKS3‐1 for KW and KT, and qKS4‐1 for KL and KT (Table S9), implicating that a close genetic correlation existed among different kernel traits in maize.

### Candidate genes co‐localized by joint linkage mapping and GWAS

Generally, genetic loci co‐localized in different genetic backgrounds were thought to have stable effects on phenotypes (Vikram *et al*., 2011). Therefore, we also focused on these genetic loci that were co‐detected in the two populations. According to the previous study (Lu *et al*., 2010), we lowered the threshold of *P*‐value to 1.0 × 10^−3^ to identify the stable loci across the two populations. Based on the physical positions of the identified QTL and SNPs, a total of 56 SNPs were found to fall in 18 of the kernel size‐related QTL (Table S10). To provide candidate genes of these co‐localized SNPs, we scanned 220‐Kb regions upstream and downstream of the 56 co‐localized SNPs based on the LD value for obtaining the genes whose orthologs/homologs in plants have been shown to regulate seed development. A total of 50 candidate genes were gained, including transcription factors, enzymes and transporters (Table S11). Interestingly, we also identified seven maize miRNAs falling within the scanned regions, including zma‐miR164e, zma‐miR169a, zma‐miR159c, zma‐miR171 l, zma‐miR319b, zma‐miR399c and zma‐miR399f (Table S11). In *Arabidopsis*, miR319, miR164, miR159, miR169 and miR171 have been demonstrated to functionally regulate the development of leaf, inflorescence, seed, root and chlorophyll biosynthesis, respectively (Koyama *et al*., 2017; Ma *et al*., 2014; Mallory *et al*., 2004; Sorin *et al*., 2014; Zhao *et al*., 2018). However, zma‐miR399 was reported to play an important role in low phosphate tolerance in maize by interacting with Pi deficiency‐induced long‐noncoding RNA1 (Du *et al*., 2018).

### Response of candidate genes to maize seed development

A previous study conducted a transcriptome analysis for the whole seeds of B73 from 0~38 days after pollination (DAP) with an interval of 2 days, which covered all 20 time points (Chen *et al*., 2014). To refer to the published transcriptome data which raw reads were aligned to the B73 reference genome (RefGen_v2), a total of 17 and 35 candidate genes, respectively, detected by GWAS and joint linkage mapping and GWAS were successfully converted to the B73 reference genome v.2 using the translation tool (https://www.maizegdb.org/gene_center/gene#translate/). All of the 17 genes identified by GWAS were expressed in maize seeds, with an average expression level of 0.26–95.66 reads per kilobase per million (RPKM; Table S12), of which 100% of the genes were differentially expressed during kernel development. Importantly, three candidate genes with the top significances and stable effect (Tables 2; Table S8) showed different dynamic expression patterns (Figure S6), reflecting their diverse roles in the corresponding stages of seed development. However, 29 (82.86%) genes detected by co‐localized SNPs showed an average expression of 0.05–28.29 RPKM in developing maize seeds, with 27 (93.10%) genes differentially expressed (Table S12). The results above indicated that the majority of these candidate genes responded to the development of maize seeds.

### Overexpression of zma‐miR164e in *Arabidopsis thaliana* down‐regulated target genes and affected grain yield

**Figure 4:**
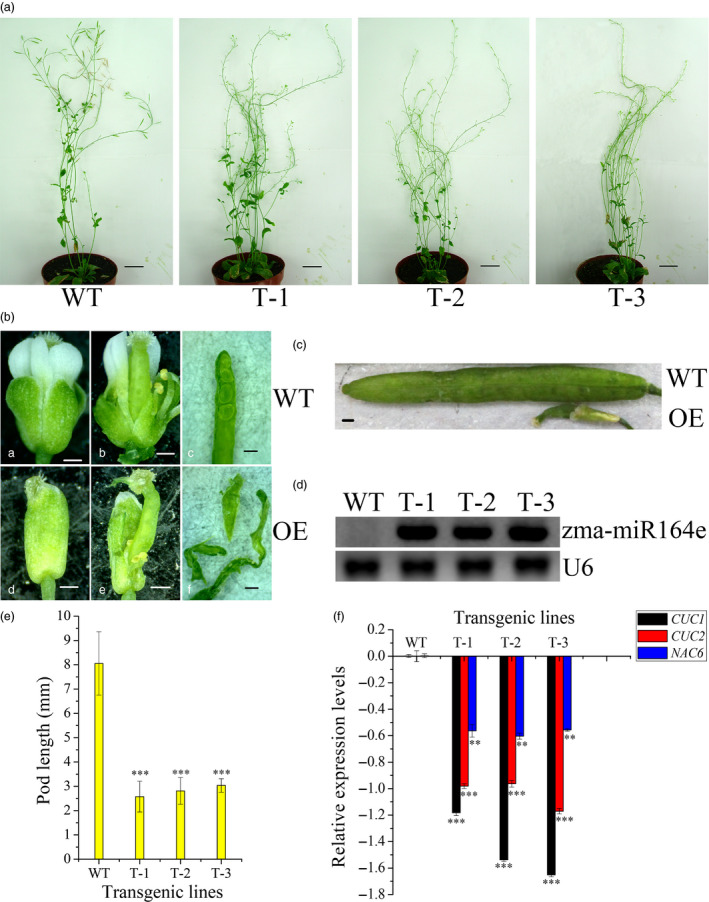
Expression of zma‐miR164e results in the failure of seed formation in *Arabidopsis*. (a) Morphological characterization of expressing zma‐miR164e (OE) transgenic plants in *Arabidopsis thaliana*. Representative plants from T1‐positive transgenic lines are shown: T‐1, T‐2 and T‐3. Wild type (WT) as the control. Bar = 2 cm. (b) The flowers and pods of the OE and WT of *Arabidopsis*. For OE, flower has no petals (d and e), pod has no seed (f); however, flower (a and b) and pod (c) of WT were normal development. Bar = 400um. (c) The mature pod of OE was significantly smaller than that of WT. Bar = 400 μm. (d) RT‐PCR analysis of the expression levels of zma‐miR164e in the mixed samples of inflorescence and buds of WT and the transgenic *Arabidopsis* plants. zma‐miR164e was no expression in WT, but expressed in the transgenic plants T‐1, T‐2 and T‐3. U6 was used as loading control. (e) The phenotypic values of pod length (mm). The pod length of the transgenic *Arabidopsis* plants was significantly shorter than WT (error bars indicate ± SD. *t*‐test; ***, *P* < 0.001). (f) qRT‐PCR analysis of *CUC1*, *CUC2* and *NAC6* in the mixed samples of inflorescence and buds of WT and the transgenic *Arabidopsis* plants. *ß‐Tubulin* was used as internal reference to calculate the relative expression of target genes using the formula log10[2^−(Ct target gene − Ct ß‐Tubulin)^]. qRT‐PCR data represent the average of three biological replicates. (Error bars indicate ± SD, *t*‐test; ***P < 0.001; **P < 0.01).

**Figure 5:**
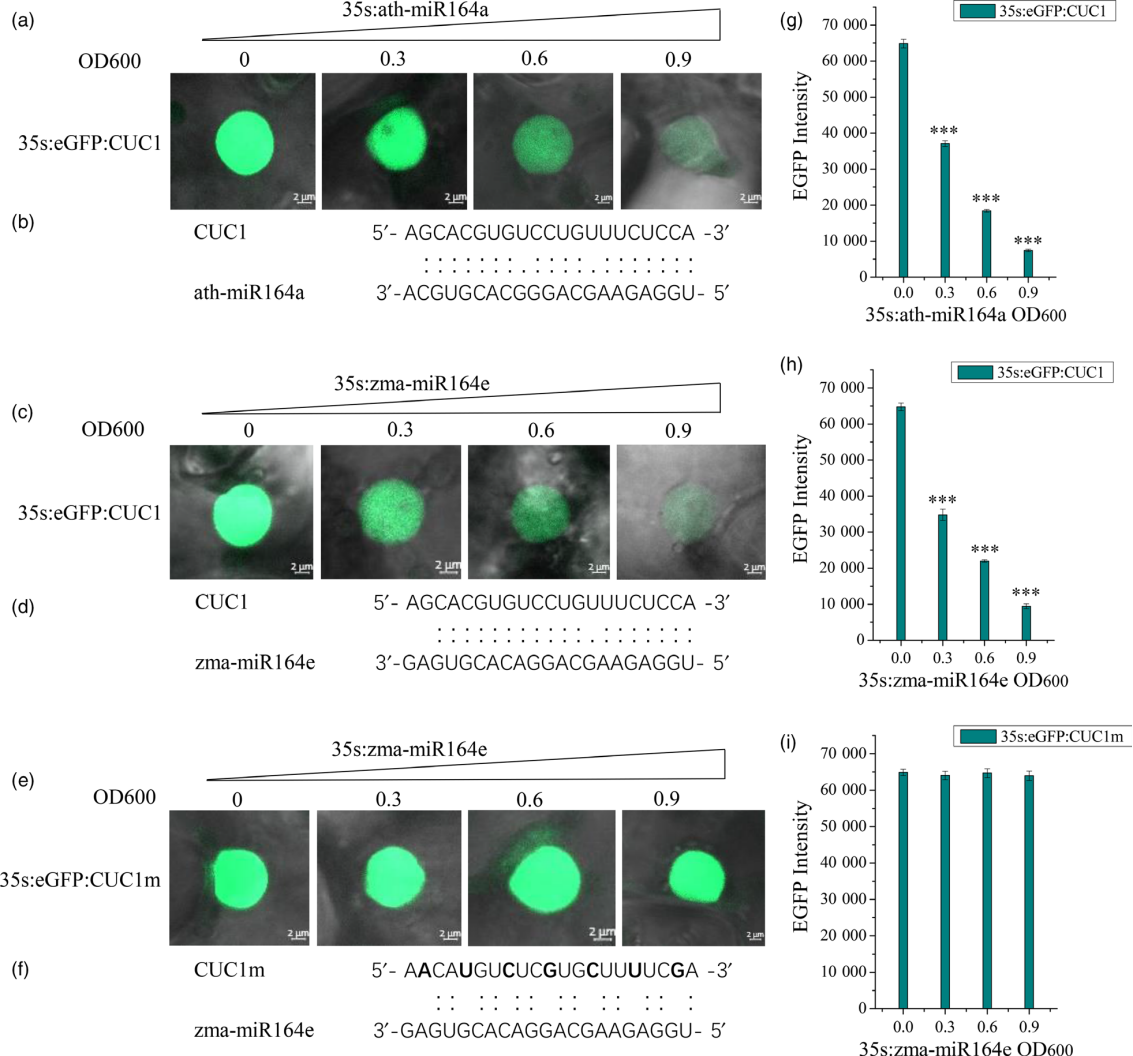
Zma‐miR164e‐directed cleavege *in Arabidopsis* *CUC1* mRNA decreases the accumulation of the CUC1 protein. (a) eGFP:CUC1 (OD_600 nm_ = 0.6) was transiently expressed alone or co‐expressed with ath‐miR164e (OD_600 nm_ = 0.3/0.6/0.9) in tobacco leaf cells. The eGFP:CUC1 protein accumulation decreased with increasing ath‐miR164a concentrations. The result served as positive control for this experiment. (b) Binding sites of ath‐miR164a and *CUC1*. (c) The eGFP:CUC1 (OD_600 nm_ = 0.6) was transiently expressed alone or co‐expressed with zma‐miR164e (OD_600 nm_ = 0.3/0.6/0.9) in tobacco leaf cells. The eGFP:CUC1 protein accumulation decreased with increasing zma‐miR164e concentrations. d, Binding sites of zma‐miR164e and *CUC1*. (e) That zma‐miR164e cannot suppress the protein accumulation of eGFP:CUC1m whose binding site sequence has synonymous mutations. (f) synonymously mutated sequence of binding sites in *CUC1*. (h) and (i) eGFP intensity change of eGFP:CUC1 and eGFP:CUC1m with the increase in zma‐miR164e concentration. (g) eGFP intensity change of eGFP:CUC1 with increasing ath‐miR164a concentrations. The data of each sample were the average of the randomly detected 10 nuclei. Same results were obtained in three independent experiments. (Error bars indicate ± SD, *t*‐test; ***P < 0.001).

Among these candidate miRNAs involving in kernel size, zma‐miR164e and zma‐miR159c had higher expression levels than the other miRNAs, which were both differentially expressed during the development of maize kernels (Li *et al*., 2016). Of them, ath‐miR159 has been previously proven to regulate the development of endosperm in *Arabidopsis* (Zhao *et al*., 2018). To further verify the function of zma‐miR164e, we expressed zma‐miR164e in *Arabidopsis thaliana* and obtained three positive transgenic lines (T1). The expression level of zma‐miR164e was confirmed using RT‐PCR, which indicated the successful expression in the three transgenic lines relative to the wild type (WT; Figure 4D). The positive transgenic plants (Figure 4A) displayed an average increase in 14 branches compared with WT, whereas no significant difference in plant height was observed between the transgenic lines and the WT. The flowers of the WT showed normal petals; however, the flowers of the transgenic plants had no petals (Figure 4Bde). More importantly, the pods of the transgenic lines were thinner and shorter (Figure 4C, E) and did not produce seeds (Figure 4Bf), indicating that the expressed zma‐miR164e affected *Arabidopsis* seed formation. Since the T1 transgenic plants failed to produce normal seeds, phenotypic investigation using biological replicates could not be performed on the T2 transgenic plants. Instead, we further conducted another two transformation experiments, which indicated that the phenotypes of the transgenic plants were similar to those in the first experiment. As the sequence of zma‐miR164e is different from any member of miR164 family in *Arabidopsis* (Figure S3), we first predicted the candidate target genes of zma‐miR164e in *Arabidopsis* using a plant small RNA target analysis site psRNATarget. The results showed that *CUC1*, *CUC2* and *NAC6* had the lowest mismatch scores (Table S13), which were then selected as the potential target genes of zma‐miR164e and were further verified by *in vitro* cleavage. Figure 5C and H shows that the fluorescence intensity of CUC1:eGFP decreased with increasing concentration (from OD_600 nm_ = 0 to OD_600 nm_ = 0.9) of zma‐miR164e in the cells of tobacco leaf co‐transformed with zma‐miR164e and CUC1:eGFP, which was similar to the positive control (Figure 5A, G). However, no change in fluorescence intensity was observed in the tobacco leaf co‐transformed with zma‐miR164e and mutated CUC1 (CUC1m):eGFP (Figure 5E, I), with increasing zma‐miR164e concentration (from OD_600 nm _= 0 to OD_600 nm_ = 0.9). These findings indicated that zma‐miR164e specifically cleaved the predicted target sequence of the *CUC1* mRNA and suppressed the accumulation of the *CUC1* protein, and the sequence change of the target region caused the failure of zma‐miR164e cleavage on the mutated *CUC1* mRNA and led to the accumulation of the *CUC1* protein. Similarly, the mRNAs of *CUC2* and *NAC6* were separately demonstrated to be cleaved by zma‐miR164e (Figures S4 and S5).

To validate the influence of the expressed zma‐miR164e on the expression of the three target genes above, qRT‐PCR was performed to determine the expression levels of these target genes in the transgenic *Arabidopsis*. The results showed that the expression levels of *CUC1*, *CUC2* and *NAC6* were all significantly lower in the transgenic lines than those in the WT (Figure 4F) in the mixed samples of inflorescence and buds. To determine whether the overexpression of zma‐miR164e triggered the up‐regulation of *Arabidopsis* miR164 family and consequently resulted in the down‐regulation of these target genes, we performed qRT‐PCR for each member (ath‐miR164a/b and ath‐miR164c) of ath‐miR164 family in the transgenic *Arabidopsis*. As shown in Figure S7, the expression levels of ath‐miR164a/b and ath‐miR164c in transgenic plants were not significantly different from those in WT. It suggested that the observed phenotypes of the transgenic *Arabidopsis* were caused by the expression of the exogenous zma‐miR164e rather than the endogenous ath‐miR164 in *Arabidopsis*. Taken together, these findings indicated that the expressed zma‐miR164e in *Arabidopsis* down‐regulated the expressions of *CUC1*, *CUC2* and *NAC6* by cleaving their mRNAs, resulting in the failure of seed production.

## Discussion

### Joint linkage mapping and association analysis is an effective method for analysing the genetic architecture of maize kernel traits

Crop yield is a complex quantitative trait. Understanding the genetic structure of maize yield contributes to high‐yield breeding in maize. QTL mapping and GWAS are both effective tools for analysing the genetic structure of quantitative traits. QTL mapping is often used to efficiently identify the chromosomal regions controlling crop agronomic traits. GWAS facilitates the identification of quantitative trait nucleotides (QTNs) and candidate genes associated with the target traits. However, QTL mapping is based on linkage analysis with biparental populations, which shows insufficient genetic diversity, and many genetic loci would therefore be lost. The main limiting factor for GWAS is the influence of the relationship of the association panel, which leads to the identification of false associations (Yu and Buckler, 2006; Yu *et al*., 2006). Furthermore, in many cases, alleles are rare from diverse germplasm collections in association populations, which severely limit the ability of GWAS to detect QTL (Lu *et al*., 2010). Therefore, a combination of linkage and association mapping can significantly improve mapping efficiency for quantitative traits.

In this study, we utilized linkage and association mapping to detect QTL and candidate genes underlying grain yield in maize. By performing GWAS using the association panels, including 310 inbred lines with 39,354 SNP markers, we obtained 21 top significant SNPs (*P* < 2.25 × 10^−6^) that were significantly associated with three kernel size traits in maize. For QTL mapping, the IBM Syn10 DH population with a higher genetic resolution than F2 and RIL populations and long genetic map length and high‐density linkage marker is more suitable for QTL fine mapping of important traits (Holloway *et al*., 2011; Liu *et al*., 2015). In the present study, we conducted QTL analysis using the IBM Syn10 DH population including 265 lines and 6,618 bin markers and identified 50 QTL controlling the three kernel size traits of maize. The physical intervals of 32 of the 50 identified QTL were within 2 Mb, which was equivalent to fine mapping. A total of 56 identified SNPs by GWAS were located in 18 of the QTL mapped in the present study (Table S10). Therefore, these 18 QTL may be utilized in the development of molecular markers for high‐yield breeding in maize.

Some QTL controlling maize kernel size were previously detected by linkage mapping or association analysis using multiple populations. For example, Liu *et al*. (2017a) identified 213 QTL for maize kernel size traits using 10 RIL populations (Liu *et al*., 2017a). Zhang *et al*. (2017) detected 24 QTL that were related to kernel size traits using RILs with an ultra‐high‐density bin map (Zhang *et al*., 2017a). Liu *et al*. (2014b) obtained 40 QTL controlling kernel size traits by linkage mapping with an F2 population derived from a cross between two maize elite inbred lines (Liu *et al*., 2014b). To distinguish the novel QTL detected by this study from the common QTL across different studies, we compared the physical genome regions between these QTL identified in our study and the previously reported QTL. If the confidence interval of a QTL identified by the present study overlapped with the QTL detected in previous studies, it was taken as a common QTL; otherwise, it was considered a novel QTL. A total of 29 QTL found in our study were common and the remaining 21 QTL are novel. It suggested that the QTL for objective traits present population common and specific characteristics (Liu *et al*., 2017a). Therefore, combining multiple populations from diverse genetic backgrounds is efficient to comprehensively analyse the genetic architecture of kernel size traits.

### Stable QTL provide important information for high‐yield maize breeding

Currently, yield stability is one of the breeding objectives in high‐yield breeding. Therefore, the identification of common QTL controlling yield across multiple environments is especially significant. In previous studies, a set of QTL has been identified to regulate yield‐related traits, most of which were located in bins 4.08, 6.05, 6.06, 7.04 and 8.06 on genomic regions and controlled kernel weight grain yield, kernel length, starch content, ear diameter, kernel row number and ear length, respectively (Portwood *et al*., 2018). These QTL were detected under Hubei, Chongqing, Henan, Yunnan, Hainan, Wuhan and Beijing. In this study, qKT4‐3, qKL6‐1, qKT6‐1, qKW7‐3 and qKW8‐1, which are located in bins 4.08, 6.05, 6.06, 7.04 and 8.06 genomic regions, were separately identified in Sichuan, Yunnan and Xinjiang environments (Table S9). In addition, seven other stable QTL (qKS5‐2, qKW10‐2, qKS1‐1, qKS4‐1, qKL3‐2, qKW7‐1 and qKT4‐2) were all identified in at least three of the seven environments in the present study. Therefore, these QTL denote the environmentally stable genetic loci underlying maize high yield and should be utilized in further fine mapping and MAS of high‐yield breeding. Notably, among these the stable QTL, qKT4‐2, had the phenotypic effect greater than 10% in two environments (10.29% in E4b and 15.04 in E5b), which was considered as a stable major QTL and worth greater emphasis.

### Candidate genes involved in kernel development

Based on the detected 21 top significant SNPs (*P* ≤ 2.25 × 10^−6^) by GWAS, 17 candidate genes were identified as potential genes regulating grain development in maize (Table 2). Interestingly, the SNP markers PZE‐101129119 and PZE‐101108339 that were detected by two models (GAPIT and TASSEL; GAPIT and FarmCPU) were located in the gene Zm00001d030895 and an intergenic region, respectively. Zm00001d030895 was annotated as adenine phosphoribosyltransferase 1 chloroplastic, which was previously reported to be first up‐regulated and then down‐regulated in developing seeds, with the highest expression at 10 DAP (Figure S6A; Chen *et al*., 2014). In *Arabidopsis*, adenine phosphoribosyltransferases (APTs) have been proven to contribute to cytokinin metabolism (Allen *et al*., 2002). Interestingly, cytokinins play an important role in the regulation of grain size, possibly resulting from cytokinins influencing accumulation processes and the duration of the filling period in barley (Michael and Seiler‐Kelbitsch, 1972). In rice, cytokinins participate in the regulation of the grain‐filling pattern during the early development of grains and affect the filling percentage of grains (Yang *et al*., 2000). Thus, Zm00001d030895 may be a novel functional gene that regulates grain size by affecting the accumulation of cytokinin in maize. In addition, the homologous genes of five candidate genes (Zm00001d025152, Zm00001d049979, Zm00001d004898, Zm00001d034507 and Zm00001d028771) have been reported to participate in the regulation of grain development (Du *et al*., 2014; Fiume and Fletcher, 2012; Li *et al*., 2015; Song *et al*., 2007; Sun *et al*., 2015), Zm00001d025152 encodes the pentatricopeptide repeat‐containing (PPR) protein. Plant PPR proteins are a large RNA‐binding protein family that regulates RNA metabolism in chloroplasts and mitochondria in plants. A number of PPR proteins (AC212684.3_FG012, GRMZM2G070381, GRMZM2G041231, GRMZM2G110851, GRMZM2G021319, GRMZM2G345128, GRMZM2G060516, GRMZM2G078416 and GRMZM2G353301) have been shown to be related to maize seed development (Cai *et al*., 2017; Dai *et al*., 2018; Li *et al*., 2018b; Qi *et al*., 2017; Ren *et al*., 2017; Sun *et al*., 2015; Xiu *et al*., 2016; Yang *et al*., 2017; Zhang *et al*., 2017b). Zm00001d049979 was annotated as embryo defective 2733, and several homologous genes of Zm00001d049979 in maize such as embryo defective 12 (GRMZM2G119691), embryo defective 14 (GRMZM2G384293), embryo defective 16 (GRMZM2G155662) and embryo defective 8516 (GRMZM2G136559) have been demonstrated to participate in the regulation of embryogenesis and seed formation (Li *et al*., 2015; Magnard *et al*., 2004; Shen *et al*., 2013; Zhang *et al*., 2013). GW2 encodes a RING‐type protein with E3 ubiquitin ligase activity and negatively regulates cell division, resulting in reduced grain width, weight and yield (Song *et al*., 2007). Zm00001d004898 was annotated as E3 ubiquitin‐protein ligase HRD1A, which is also a RING zinc finger domain superfamily protein, and thus may have the similar function of regulating grain development. Zm00001d034507 was annotated as CLAVATA3/ESR‐related protein 25, the homologous gene of which *CLE8* regulates embryo and endosperm development in *Arabidopsis* (Fiume and Fletcher, 2012). Zm00001d028771 was annotated as ubiquitin‐activating enzyme E12, which participates in the ubiquitin–proteasome pathway (Salceda and Caro, 1997). The ubiquitin–proteasome pathway has been reported to be important in controlling plant seed development (Disch *et al*., 2006; Du *et al*., 2014; Li *et al*., 2008; Peng *et al*., 2015; Xia *et al*., 2013).

### miRNAs provide an effective way to improve complex yield traits in maize

Grain yield is a complex quantitative trait that is controlled by a large number of genetic factors, including protein‐coding genes and regulatory factors (Fu *et al*., 2012; Yan *et al*., 2006). As a class of small regulatory factors, plant miRNAs control growth and development and stress responses in plants by manipulating the expression of their target genes (Fu *et al*., 2012; Yan *et al*., 2006). Mature miRNAs are processed from their longer precursors, and the latter are generated from pri‐miRNAs, which are transcribed from MIR genes (Moro *et al*., 2019; Pegler *et al*., 2019). The variations in miRNA and the promoter of MIR genes have been shown to affect the phenotypes (Hommers *et al*., 2018; Sadeghi *et al*., 2018). Therefore, the identification of miRNAs regulating phenotypes is feasible through forward‐genetic approaches. In the present study, we first identified the candidate miRNAs involved in maize grain yield using a combination of QTL mapping and GWAS. Among these, five (71.43%) miRNAs have been reported to be correlated with growth and development in plants. Notably, ath‐miR159 has been previously reported to regulate the development of endosperm in *Arabidopsis* (Zhao *et al*., 2018). In addition, miR164 is a conservative miRNA family in plants, which has five NAC domain target genes, including *NAC1*, *CUC1*, *CUC2*, *At5 g07680* and *At5 g61430*, and mainly regulates lateral root development, meristem development and flower organ development in *Arabidopsis* (Guo *et al*., 2005; Sieber *et al*., 2007). Guo *et al*. (2005) reported that miRNA164 directs mRNA cleavage of the transcription factor *NAC1* to down‐regulate auxin signals for *Arabidopsis* lateral root development (Guo *et al*., 2005). Mallory *et al*. (2004) and Laufs *et al*. (2004) have independently revealed that miR164 cleaves target genes *CUC1* and *CUC2* to regulate meristem development and flower organ development (Laufs *et al*., 2004; Mallory *et al*., 2004). The dynamic expression pattern of miRNAs during maize kernel development showed that the miR164 family responds to embryogenesis and seed development in maize (Li *et al*., 2016). Liu *et al*. (2014) provided evidence that miR164 participates in the development of maize ears (Liu *et al*., 2014a).

In this study, the overexpression of zma‐miR164e in *Arabidopsis* down‐regulated the expression levels of *CUC1*, *CUC2* and *NAC6*, resulting in the increased branch number and the failure of generating normal seeds, thereby influencing kernel yield of *Arabidopsis*. Moreover, we also used the sequence of zma‐miR164e to predict its target genes in maize, which indicated that *Zm00001d016950* (*NAC30*), *Zm00001d041472* (*NAC108*) and *Zm00001d014405* (*NAC113*) have the highest expectations. Interestingly, each of the above genes showed a high expression level in maize flowers and seeds (Walley *et al*., 2016; Wang *et al*., 2018), and two of their orthologs (*Arabidopsis CUC1* and *CUC2*) have been previously reported to participate in embryo formation in *Arabidopsis* (Mallory *et al*., 2004 *)*. Combined these findings suggested that zma‐miR164e regulates grain yield in maize and that miR164 has a conserved function on seed development between maize and *Arabidopsis*.

## Experimental procedures

### Materials and phenotypic evaluation

The association panel consisted of 310 inbred lines, which were collected from the breeding programme of Southwest China, as described by Zhang *et al*. (2016). These lines were planted in a randomized complete block design in two replicates. Each plot contained a single row (14 plants) that was 3 m in length and 0.75 m from the next row, and the planting density was ~62,000 individuals per hectare. Each line was grown in a single row. These field traits for GWAS were measured in three environments in 2016, including Xishuangbanna (Jinghong, E100°46′, N22°0′), Sichuan (Hongya, E103°22′, N29°55′) and Sichuan (Ya'an, E103°0′, N29°59′), which were designated as E1a, E2a and E3a, respectively. Best linear unbiased prediction (BLUP) values of each trait in all of the above environments were used to represent the phenotypic values for E4a. The three kernel size‐related traits included kernel length (KL, cm), kernel width (KW, cm) and kernel thickness (KT, mm), among which KL and KW were examined in each individual by randomly selecting 10 kernels from the centre of each ear, whereas KT was examined using a total of five kernels. Five maize ears with good self‐pollination were selected to evaluate the phenotypes of each line, two sets of data for each trait were obtained for each ear, and each population was planted with two replicates per environment. The KW and KL of 10 seeds were measured using a ruler with a precision of 0.05 mm, and KT of five seeds was measured using an electronic digital calliper with a precision of 0.01 mm. The average value of two replicates of each trait per line in a single environment was calculated to represent trait performance in association analysis (Table S1).

A total of 265 lines from the IBM Syn10 DH population were used for QTL analysis of various kernel size traits. There were highly significant differences on the investigated traits between the two parents in the multiple environments (Figure 1). Mo17, which had large seeds, had an average KL of 10.85 mm, an average KW of 9.26 mm and an average KT of 4.87 mm across multiple environments. In contrast, B73, which had smaller seeds, had averages of 9.10, 5.35 and 3.81 mm for KL, KW and KT, respectively. KL and KW of the IBM population were evaluated in six environments in China, including Sichuan (Chongzhou, E103°40′, N30°40′) in 2016 and 2017, Sichuan (Wenjiang, E103°50′, N30°42′) in 2016 and 2017, Xishuangbanna (Jinghong, E100°46′, N22°0′) in 2016 and Xinjiang (Changji, E87°18′, N44°01′) in 2017. KT was evaluated in three of the above environments (Table S2). These location/year combinations were designated as E1b (Chongzhou, 2016), E2b (Wenjiang, 2016), E3b (Jinghong, 2016), E4b (Chongzhou, 2017), E5b (Wenjiang, 2017) and E6b (Changji, 2017). The way of investigating these traits and the field experiment design for the IBM population were the same as those for the association pool described above except for KT, which was measured using a ruler with a precision of 0.05 mm for 10 seeds (Table S2).

### Phenotypic data analysis

Descriptive statistical analysis of the phenotypic data and correlation analysis between the traits were performed with IBM SPSS Statistics version 21.0 software (Kinnear, 2011). Broad‐sense heritability (H^2^) for each trait was estimated as described by Knapp (Knapp *et al*., 1985) as: H^2^ = δ^2 ^g/(δ^2 ^g + δ^2 ^ge/*n* + δ^2^/*nr*), where δ^2^g, δ^2^ge and δ^2^ are estimates of genetic variance, interaction variance of genotype × environment and error variance; and *n* and *r* are the number of environments and replications per environment, respectively. The best linear unbiased prediction (BLUP) values were computed using the R package lme4 (version 3.4.2, https://www.r-project.org/ **),** which was fitted to each phenotype (Goldberger, 1962):

$$\begin{array}{c} \text{BLUP}=\text{lmer}(\text{Phenotype}∼[\left(1|\text{Lines}\right)+\left(1|\text{Location}\right)\\ \;+\left(1|\text{Year}\right)+\left(1|\text{Repeat}\;\%\;\text{in}\;\%\;\text{Location}:\text{Year}\right)\\ \;+\left(1|\text{Lines}:\text{Location}\right)+\left(1|\text{Lines}:\text{Year}\right)]. \end{array}$$

### Genome‐wide association mapping

Using publicly available genotypic data from our previous study, all 310 of the lines of the association panel contained 56,110 SNP loci (Zhang *et al*., 2016). A total of 39,354 SNPs across 10 chromosomes remained after quality filtering using the following standard: SNPs with a missing rate >5%, SNPs with heterozygosity rate > 20% and SNPs with a minor allele frequency (MAF) <0.05 were expurgated, and only biallelic sites were reserved. The resulting 39,354 SNPs were subsequently used for LD calculation and GWAS analysis. Population structure was estimated by using STRUCTURE 2.3.4 software program with the 5,000 SNPs, which were randomly selected from the above 39,354 SNPs and evenly distributed across 10 chromosomes (Pritchard *et al*., 2000), and a Bayesian Markov chain Monte Carlo programme was utilized for assigning individuals to groups. The number of subgroups (K) was set from 1 to 10, and five‐time simulations with iterations and burn‐ins set to 10,000 were conducted using the mixture model and correlated allele frequency for each K. Based on the output log likelihood of data (LnP(D)) of STRUCTURE, the ad hoc statistic ΔK was applied to determine the optimal number of subgroups (Evanno *et al*., 2005). Principle component analysis (PCA) was also performed in R software for calculating the population structure and compared with the result of STRUCTURE. The best number of subpopulations was selected to determine the Q matrix. The software SpAGeDi (Hardy and Vekemans, 2002) was used to calculate kinship coefficients (kinship matrix) between the inbred lines of the associated panel. LD between genome‐wide SNP markers was calculated using TASSEL 5.0 software (Bradbury *et al*., 2007).

GWAS analysis for the kernel size traits was conducted using three association analysis methods to balance false positives and false negatives in the present study: (i) the compressed mixed linear model (CMLM) in GAPIT (Lipka *et al*., 2012; Zhang *et al*., 2010) with the kinship matrix and population structure (Q matrix); (ii) the mixed linear model (MLM) in TASSEL 5.0 (Bradbury *et al*., 2007) with the kinship matrix and Q matrix; and (iii) Fixed and random model Circulating Probability Unification (FarmCPU; Liu *et al*., 2016b) with population structure (PCA) as covariates. The statistical program simpleM implemented in R was used to explain the multiple testing for SNPs, and the results showed that 22,223 SNPs were effective (Johnson *et al*., 2010). Therefore, the threshold level for significant trait‐marker associations was set as 2.25 × 10^−6^ based on the effective number of SNPs and familywise error rate as α = 0.05 (Hu *et al*., 2017). The phenotypic variation explained (PVE) for SNPs identified by the FarmCPU was calculated as follows:

$$r^{2}=\frac{\sum_{i=1}^{n}{\left({\hat{y}}_{i}-\hat{y}\right)}^{2}}{\sum_{i=1}^{n}{\left(y_{i}-y\right)}^{2}},$$

where *y*_*i*_ is the observed phenotype value, and ${\hat{y}}_{i}$ is the estimated phenotype value from a multiple linear regression model that was fitted to all significant SNPs as an independent variable with fixed effect (Martinez *et al*., 2018).

### Linkage mapping

In our previous study, a bin map with 6,618 recombination bins was constructed for the IBM population (Liu *et al*., 2015). The average distance is 0.48 cM between adjacent markers. In this study, QTL controlling the kernel size in seven (KL and KW) or four (KT) of the environments (E1b, E2b, E3b, E4b, E5b, E6b and E7b) were detected using a composition‐interval mapping (CIM) method by applying Windows QTL Cartographer software version 2.5 (Wang *et al*., 2012a). The programme settings were as follows: CIM model = Model 6: Standard model; control markers numbers = 5; window size = 10 centimorgans; regression method = Backward regression method; walk speed = 0.5 centimorgan. We used an LOD = 2.5 as the threshold, and the 2‐LOD interval was considered as the QTL candidate region. When the confidence intervals of two identified QTL for a single trait or multiple traits overlapped, these were considered a single unique QTL. Among these, QTL detected for multiple traits were defined as pleiotropic QTL.

### Genetic transformation of zma‐miR164e in *Arabidopsis thaliana*

A 426‐bp fragment of the zma‐miR164e precursor was cloned from the genomic DNA of B73 with the primer pair Pre‐F and Pre‐R (Table S14). The DNA fragment was then ligated into the multiple cloning sites between the CaMV 35S promoter and the nos terminator in the plant binary expression vector pRI‐101‐AN using the In‐Fusion ligase enzyme (Clontech). The resultant 35S:pre‐miR164e plasmid was transformed to *Agrobacterium tumefaciens* strain GV3101, which was then used to transform *Arabidopsis thaliana* (Colombia) using the floral dip method (Clough and Bent, 2010). The collected seeds were surface‐sterilized and plated on ½ MS media containing 50 μg/mL kanamycin for selection of positive transformed plants. Then, the viable transgenic plants were transplanted into nutritive soil and grown under long‐day conditions (16‐h light/8‐h dark) at 22°C in a greenhouse.

### Prediction and validation of target genes of zma‐miR164e in *Arabidopsis*

The target genes of zma‐miR164e in *Arabidopsis* were predicted using a plant small RNA target analysis website (http://plantgrn.noble.org/psRNATarget/). The three genes with the lowest mismatching scores were *CUC2*, *CUC1* and *NAC6* (Table S13), which were considered the candidate target genes of zma‐miR164e. To verify zma‐miR164e‐directed cleavage in *Arabidopsis CUC1, CUC2 and NAC6 mRNAs, we* constructed six vectors, namely pCAMBIA2300‐35s:eGFP:CUC1 (V1), pCAMBIA2300‐35s:eGFP:CUC1m (V1m), pCAMBIA2300‐35s:eGFP:CUC2 (V2), pCAMBIA2300‐35s:eGFP:CUC2m (V2m), pCAMBIA2300‐35s:eGFP:NAC6 (V3) and pCAMBIA2300‐35s:eGFP:NAC6m (V3m). *Arabidopsis* (Colombia) total RNA was prepared from inflorescence and converted to cDNA for gene cloning. The full‐length *CUC1*_CDS, *CUC2*_CDS and *NAC6*_CDS sequences lacking stop codons were amplified with primer pairs *CUC1*‐F/*CUC1*‐R, *CUC2*‐F/*CUC2*‐R and *NAC6*‐F/*NAC6*‐R (Table S14) by PCR, respectively, using the cDNA above as the templates. Then, the three amplification productions (*CUC1*_CDS, *CUC2*_CDS and *NAC6*_CDS) were separately ligated between the CaMV 35S promoter and the eGFP in the fusion expression vector pCAMBIA2300‐35S‐eGFP, generating the final plasmids V1, V2 and V3, respectively. Furthermore, we generated *CUC1m*‐1, *CUC2m*‐1 and *NAC6m*‐1 through PCR by applying the mutated primer pairs (*CUC1*‐F/*muCUC1*‐R, *CUC2*‐F/*muCUC2*‐R and *NAC6*‐F/*muNAC6*‐R; Table S14) that encompassed the predicted binding sites of zma‐miR164e in *CUC1*, *CUC2* and *NAC6*, which resulted in synonymous mutations of the seven amino acids in the predicted binding sites (Figures 5F; Tables S4F and S5F). The 276, 333 and 204 bp downstream the bound sites of *CUC1*_CDS, *CUC2*_CDS and *NAC6*_CDS (*CUC1m*‐2, *CUC2m*‐2 and *NAC6m*‐2) were amplified with primer pairs *CUC1*‐F2/*CUC1*‐R, *CUC2*‐F2/*CUC2*‐R and *NAC6*‐F2/*NAC6*‐R (Table S14). The resulting fragments were predicted to fail to be targeted by zma‐miR164e and therefore called zma‐miR164e‐resistant version *CUC1*_CDS (*CUC1m*), *CUC2*_ CDS (*CUC2m*) and *NAC6*_CDS (*NAC6m*). These amplification products (*CUC1m*‐1 and *CUC1m*‐2, *CUC2m*‐1 and *CUC2m*‐2, and *NAC6m*‐1 and *NAC6m*‐2) were separately ligated between the CaMV 35S promoter and the eGFP in the fusion expression vector pCAMBIA2300‐35S‐eGFP, generating the final plasmids V1m, V2m and V3m, respectively. The plasmid 35S:pre‐miR164e and V1 were co‐transformed into tobacco leaves by *Agrobacterium* injection, as well as 35S:pre‐miR164e and V2, and 35S:pre‐miR164e and V3 using co‐transformation of 35S:pre‐miR164e and V1m, 35S:pre‐miR164e and V2m, and of 35S: pre‐miR164e and V3m as negative controls, respectively. As ath‐miR164a has been previously proven to cleave the *CUC1* in *Arabidopsis*, we used the co‐transformation of ath‐miR164a and V1 as positive control. At 48 h after Agrobacterium injection, the fluorescence intensity was assessed in these transformed tobacco leaves using a fluorescence confocal microscope (LSM 800, ZEISS, Germany), with plan‐Apochromat 40x/0.95 Korr M27, eGFP excitation/emission wavelength of 488 nm/509 nm, laser intensity of 1.00% and detector gain of 614V.

### Expression analysis of zma‐miR164e and its target genes in transgenic *Arabidopsis*

The genomic DNA of the transgenic *Arabidopsis* lines was extracted using the CTAB method, and primer pair pRI101‐F and pRI101‐R (Table S14) from T‐DNA sequence was used to confirm the positive transgenic lines. Total RNA was isolated from a mix of inflorescence and buds of the positively transgenic plants or WT plants using HiPure Plant RNA Mini Kit (Magen, Guangzhou, China). The Mir‐X miRNA first‐strand synthesis kit (Clontech, Mountain View, CA) was used for the reverse transcription of miRNAs to enable zma‐miR164e to be quantified by reverse transcription‐polymerase chain reaction (RT‐PCR). The resulting miRNAs were appended with tag sequences. The entire sequence of zma‐miR164e was used as 5′ primer, and the 3′ primer was a part of the tag sequence supplied with the kit. PrimeScript^™^RT reagent kit with a gDNA Eraser (Takara, Dalian, China) was used for converting mRNAs into cDNA for quantitative real‐time PCR to determine the expression levels of *CUC1*, *CUC2* and *NAC6* in the transgenic lines, using ß‐tubulin as internal reference. All of the primers are listed in Table S14.

## Conflict of interest

The authors declare they have no conflict of interest.

## Authors’ contributions

YS and GP designed the experiments; ML, XT, YY, PL, XZ, YZ, LW and YH conducted the experiments and performed the analysis. ML and YS drafted the manuscript. All of the authors critically revised and approved the final version of this manuscript.

## Supporting information


**Figure S1** Manhattan plots of the association analysis for KL, KW, and KT in four environments.

**Figure S2** Quantile‐quantile plots for the association study of kernel size traits in maize.

**Figure S3** The mature sequences of ath‐miR164 family members and zma‐miR164e.

**Figure S4** Zma‐miR164e‐directed cleaves *Arabidopsis CUC2* and decreases the accumulation of the *CUC2* protein.

**Figure S5** Zma‐miR164e‐directed cleaves *Arabidopsis NAC6* and decreases the accumulation of the *NAC6* protein.

**Figure S6** Expression level of three genes with the top significances and stable effect in developing seed.

**Figure S7** Expression level of each member of ath‐miR164 family in the transgenic *Arabidopsis* plants.

**Table S1** Phenotypes of the 310 maize inbred lines across three environments.
**Table S2** Phenotypes of the IBM Syn 10 DH population across six environments.
**Table S3** Phenotypic correlation coefficients between the grain traits across three environments in the association panel.
**Table S4** Phenotypic correlation coefficients between the grain traits across six environments in the IBM Syn 10 DH population.
**Table S5** Environmental correlation coefficients of three grain traits in the association panel.
**Table S6** Environmental correlation coefficients of three grain traits in the IBM Syn 10 DH population.
**Table S7** Significant SNPs with stable effect for maize kernel size traits detected by GWAS using three models (GAPIT, TASSEL and FarmCPU).
**Table S8** Candidate genes for stable‐effect SNPs significantly associated with maize kernel size traits.
**Table S9** QTL identified for maize kernel size traits across seven environments using a high‐density bin map.
**Table S10** Co‐localized SNPs and QTL combined association and linkage mapping.
**Table S11** Co‐localized candidate genes by combined association and linkage mapping.
**Table S12** Dynamic expression patterns of the candidate genes in the transcriptomes date in developing seeds.
**Table S13** Candidate target genes of zma‐miR164e in *Arabidopsis*.
**Table S14** Primer sequence used in the present study.
