# Supplementary figures and images for: Analysis of the genetic architecture of maize kernel size traits by combined linkage and association mapping
Source: Plant Biotechnol J. 2019 Jun 26;18(1):207–21. doi: 10.1111/pbi.13188 (PMC6920160; doi:10.1111/pbi.13188)

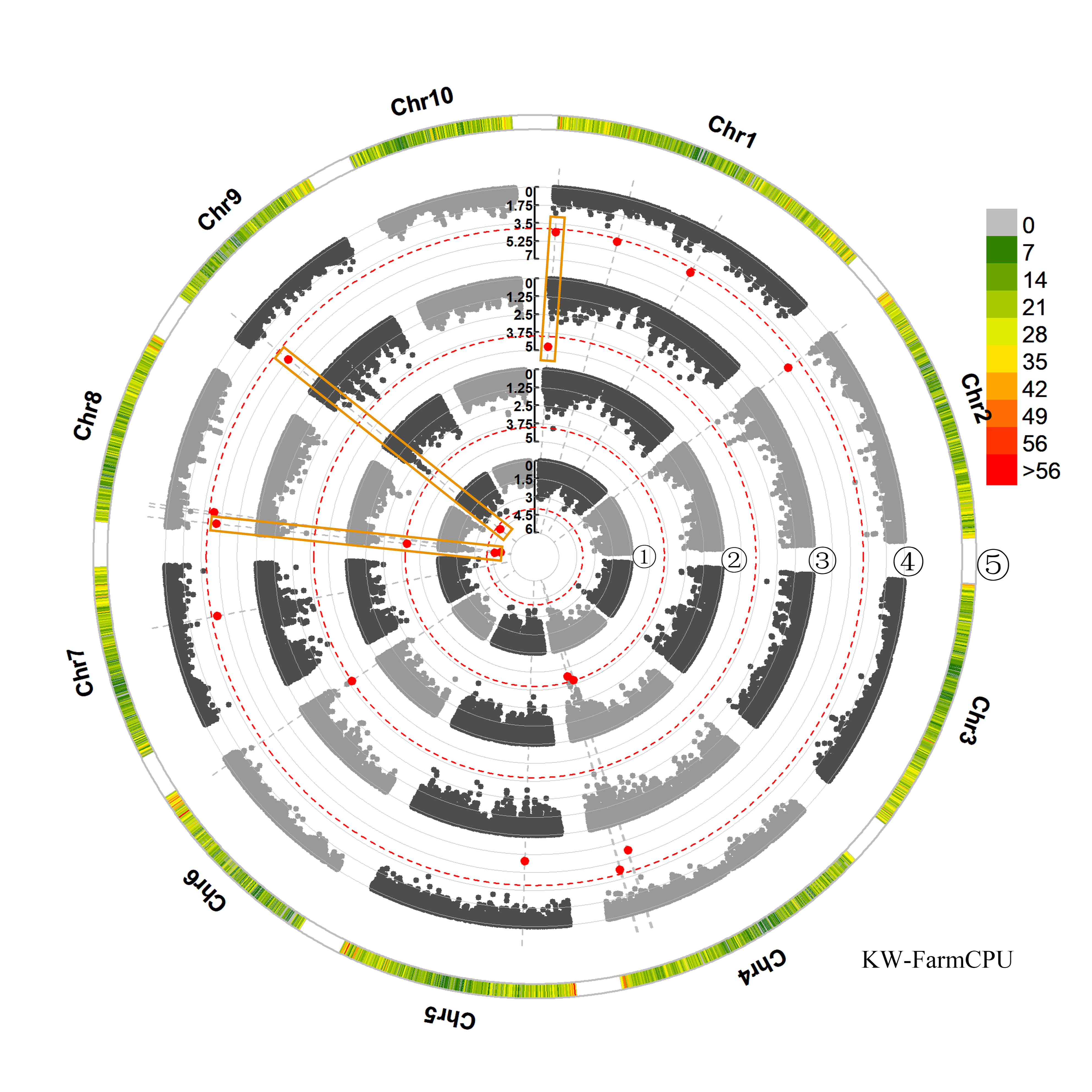

Supplement: Supplementary file 1 — Figure S1 Manhattan plots of the association analysis for KL, KW, and KT in four environments. [file PBI-18-207-s001.zip › pbi13188-sup-0001-FigS1-A.tif]

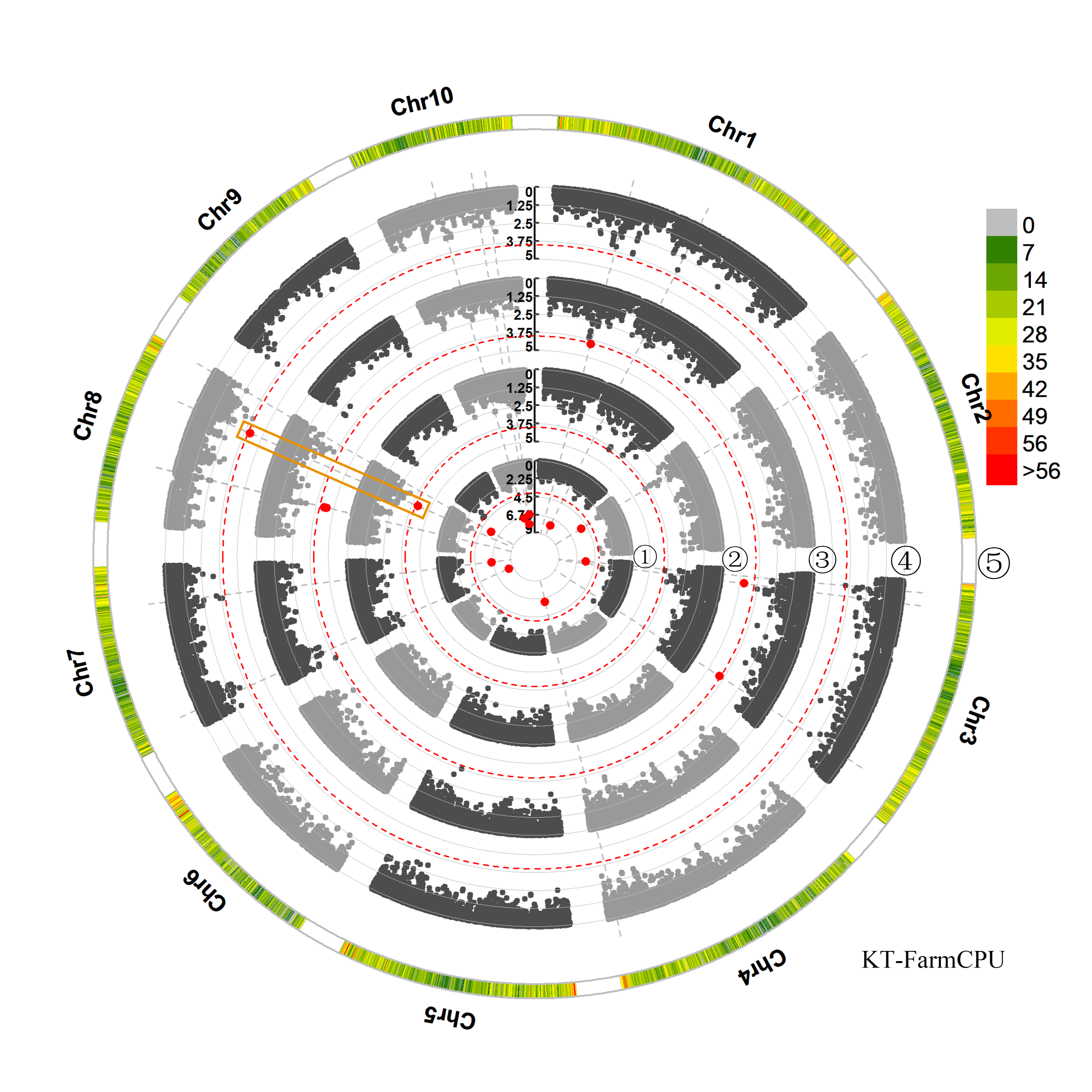

Supplement: Supplementary file 1 — Figure S1 Manhattan plots of the association analysis for KL, KW, and KT in four environments. [file PBI-18-207-s001.zip › pbi13188-sup-0002-FigS1-B.tif]

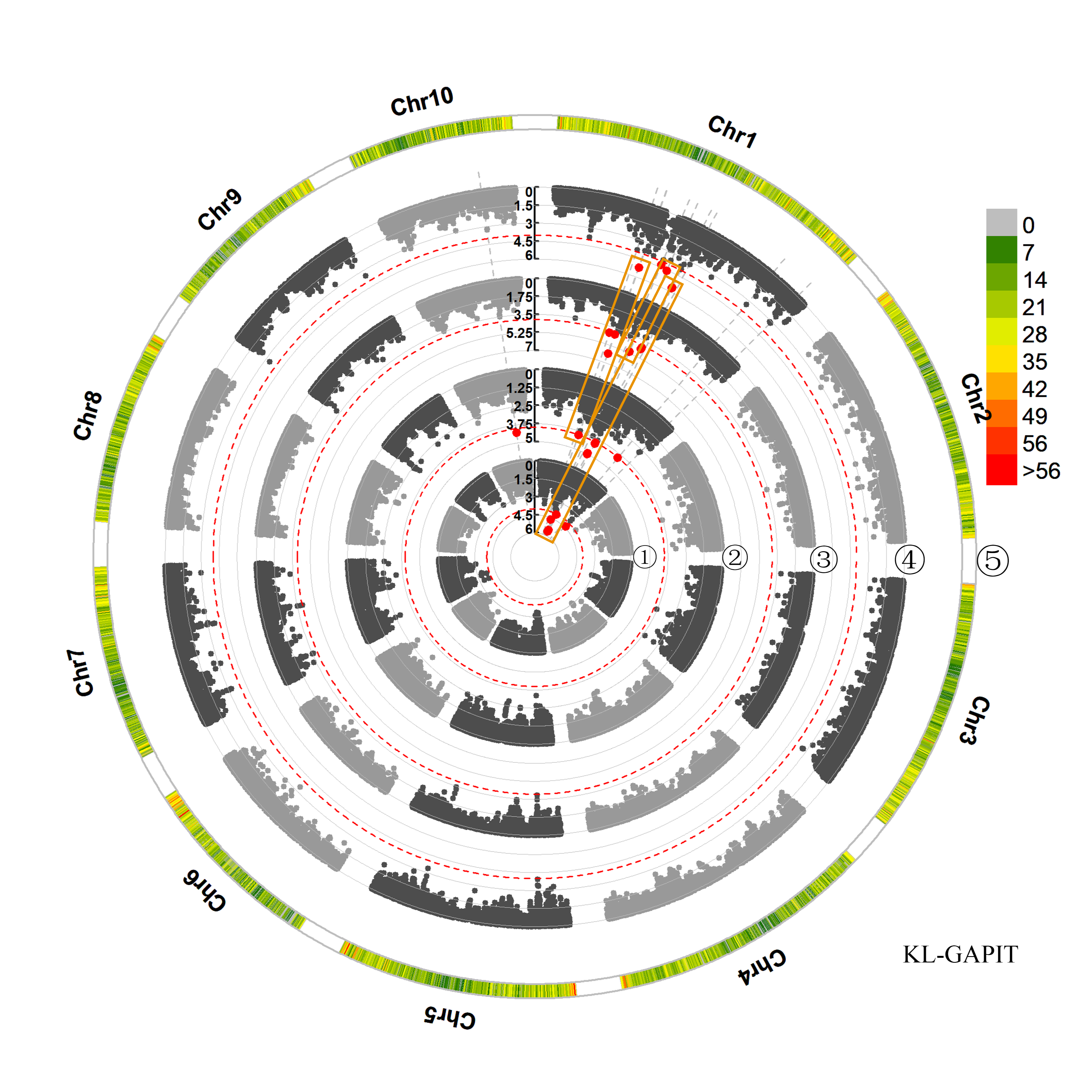

Supplement: Supplementary file 1 — Figure S1 Manhattan plots of the association analysis for KL, KW, and KT in four environments. [file PBI-18-207-s001.zip › pbi13188-sup-0003-FigS1-C.tif]

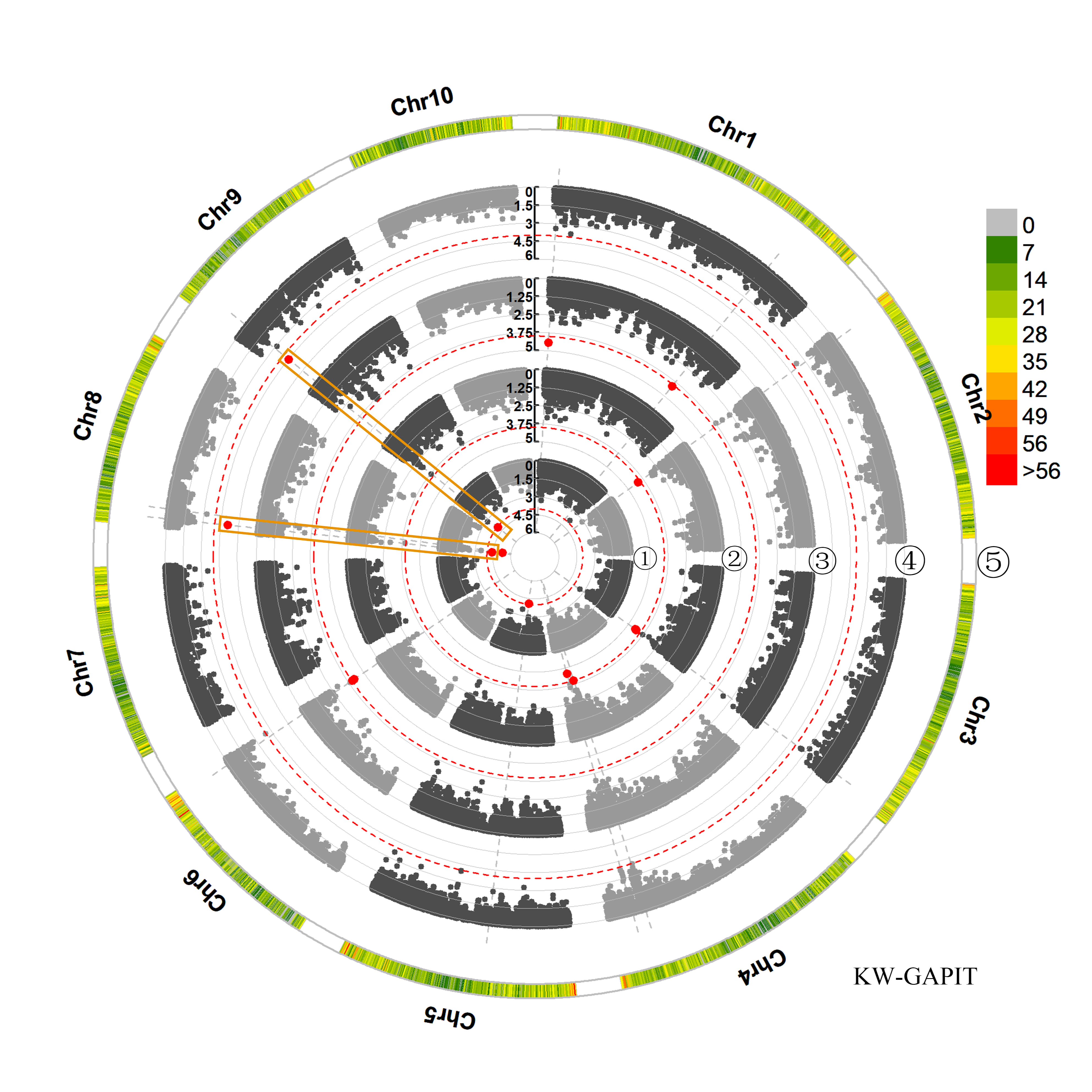

Supplement: Supplementary file 1 — Figure S1 Manhattan plots of the association analysis for KL, KW, and KT in four environments. [file PBI-18-207-s001.zip › pbi13188-sup-0004-FigS1-D.tif]

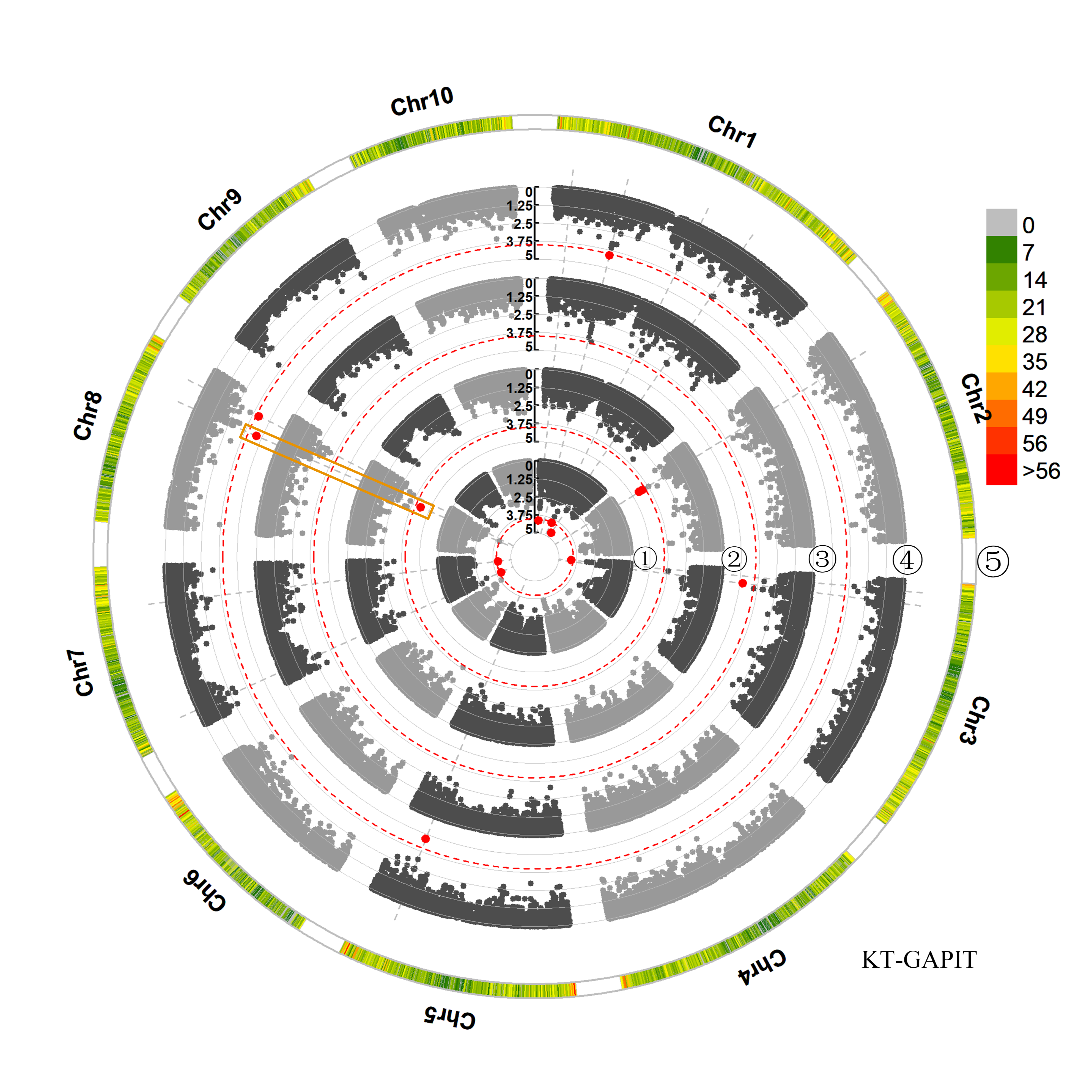

Supplement: Supplementary file 1 — Figure S1 Manhattan plots of the association analysis for KL, KW, and KT in four environments. [file PBI-18-207-s001.zip › pbi13188-sup-0005-FigS1-E.tif]

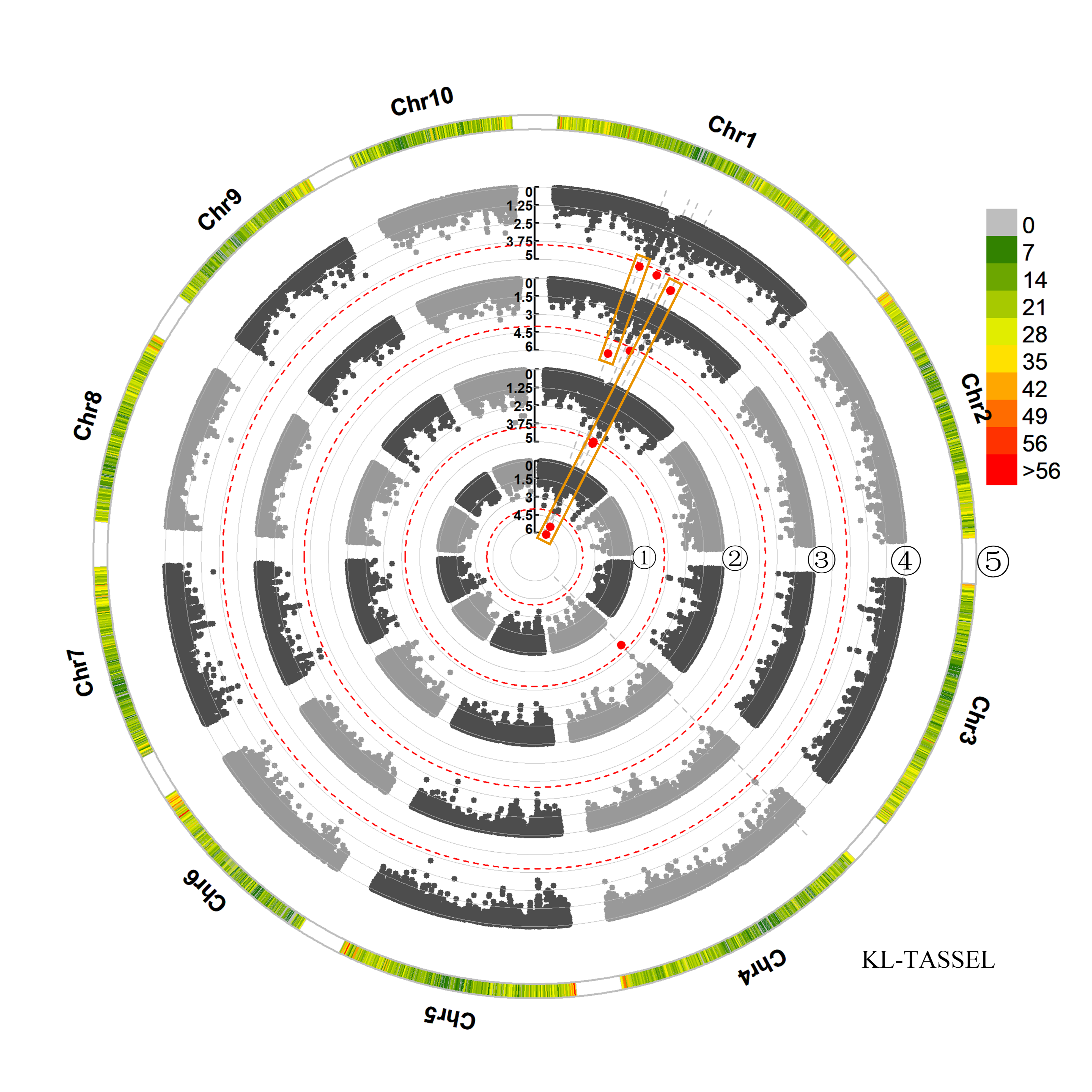

Supplement: Supplementary file 1 — Figure S1 Manhattan plots of the association analysis for KL, KW, and KT in four environments. [file PBI-18-207-s001.zip › pbi13188-sup-0006-FigS1-F.tif]

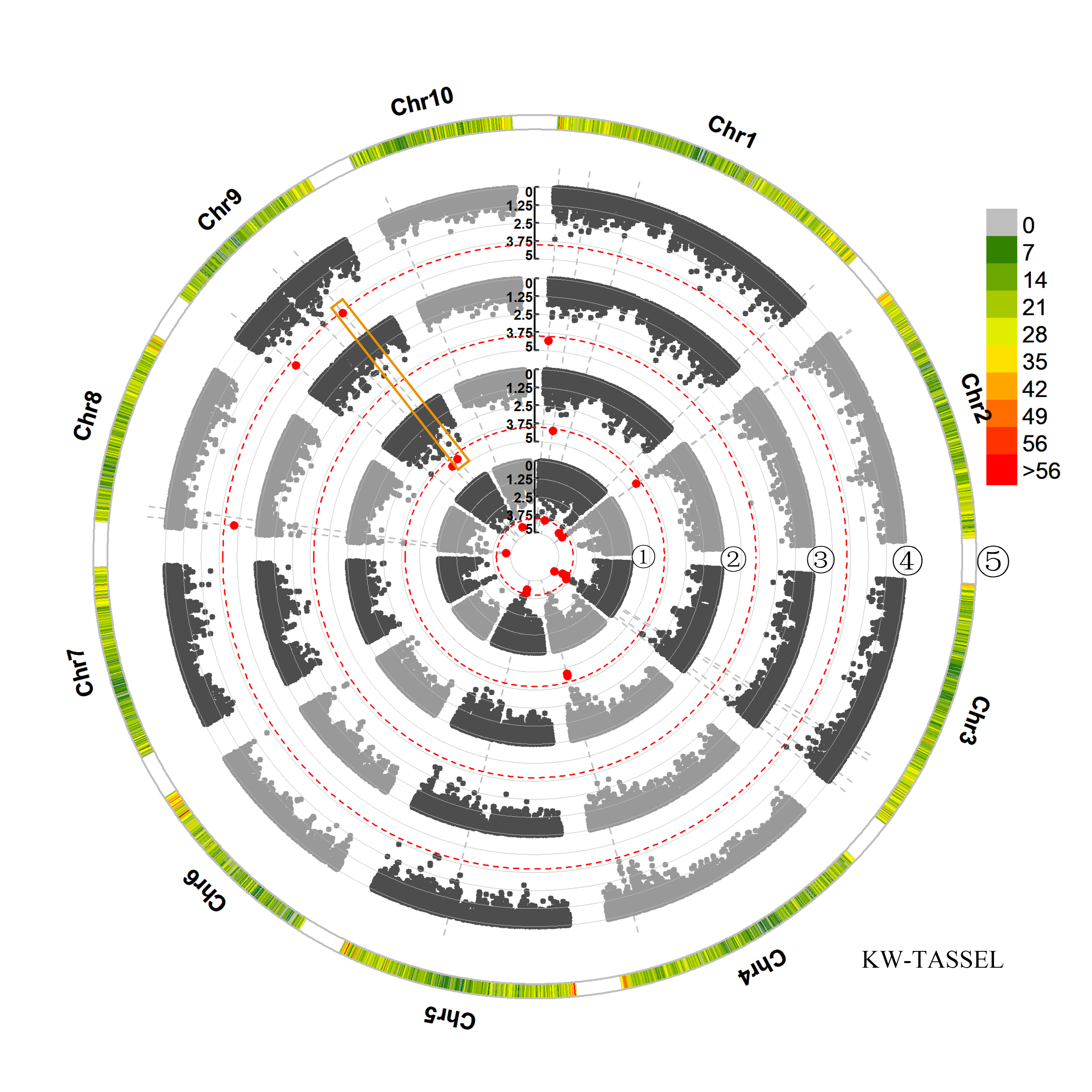

Supplement: Supplementary file 1 — Figure S1 Manhattan plots of the association analysis for KL, KW, and KT in four environments. [file PBI-18-207-s001.zip › pbi13188-sup-0007-FigS1-G.tif]

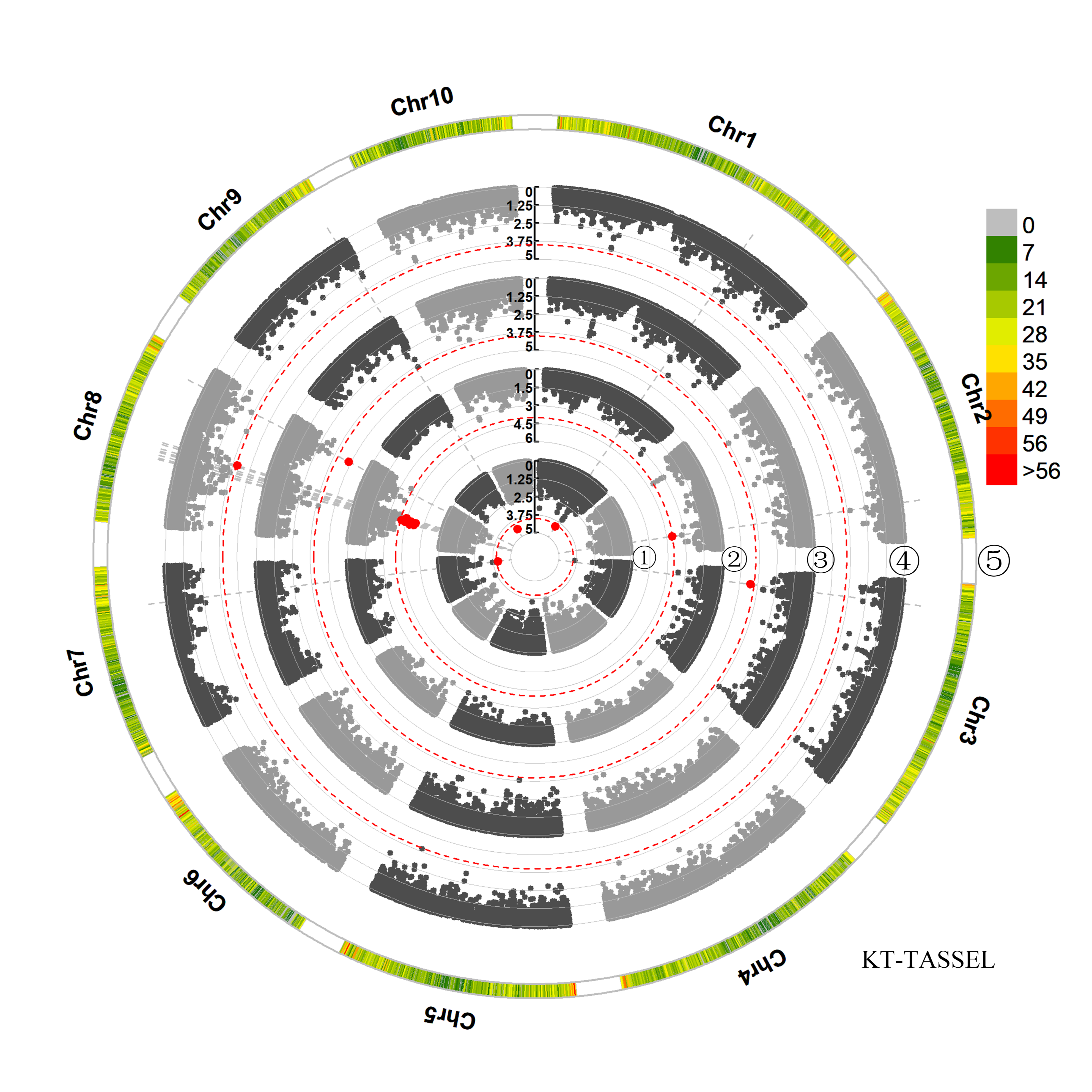

Supplement: Supplementary file 1 — Figure S1 Manhattan plots of the association analysis for KL, KW, and KT in four environments. [file PBI-18-207-s001.zip › pbi13188-sup-0008-FigS1-H.tif]

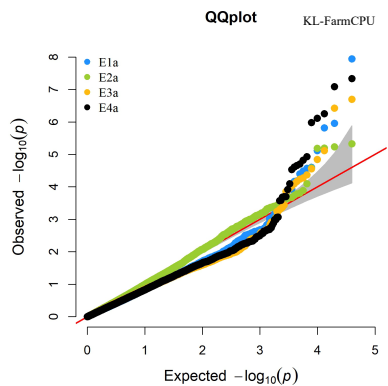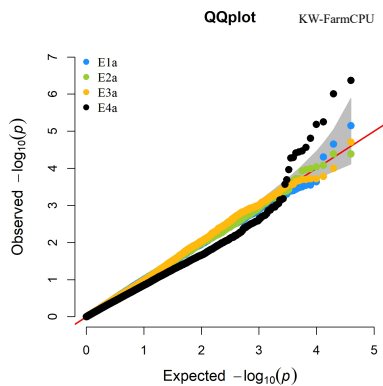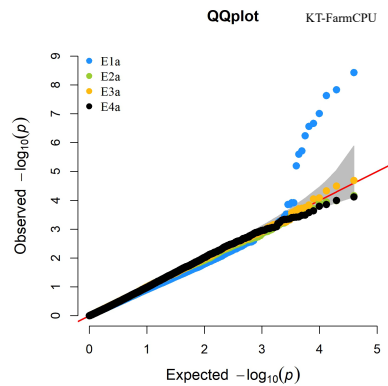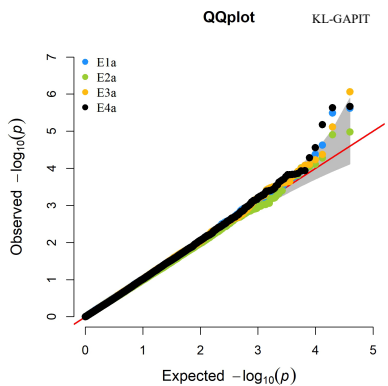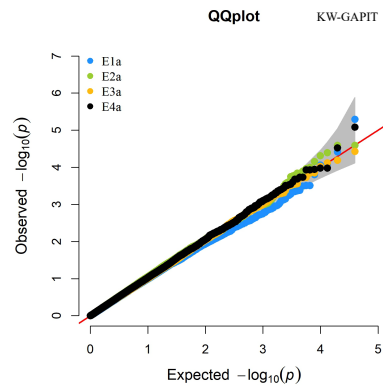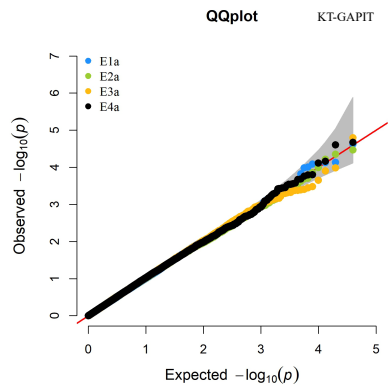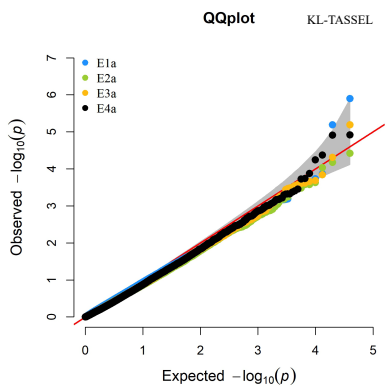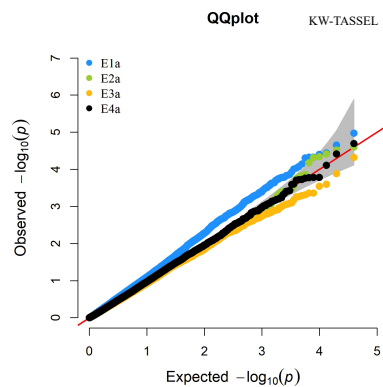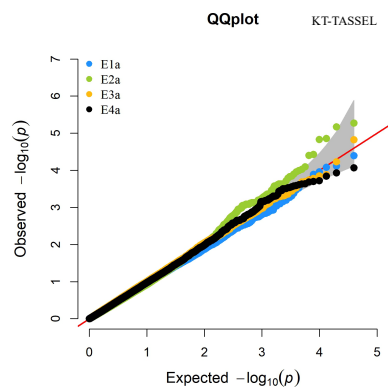

Supplement: Supplementary file 2 — Figure S2 Quantile‐quantile plots for the association study of kernel size traits in maize. [file PBI-18-207-s006.pdf]

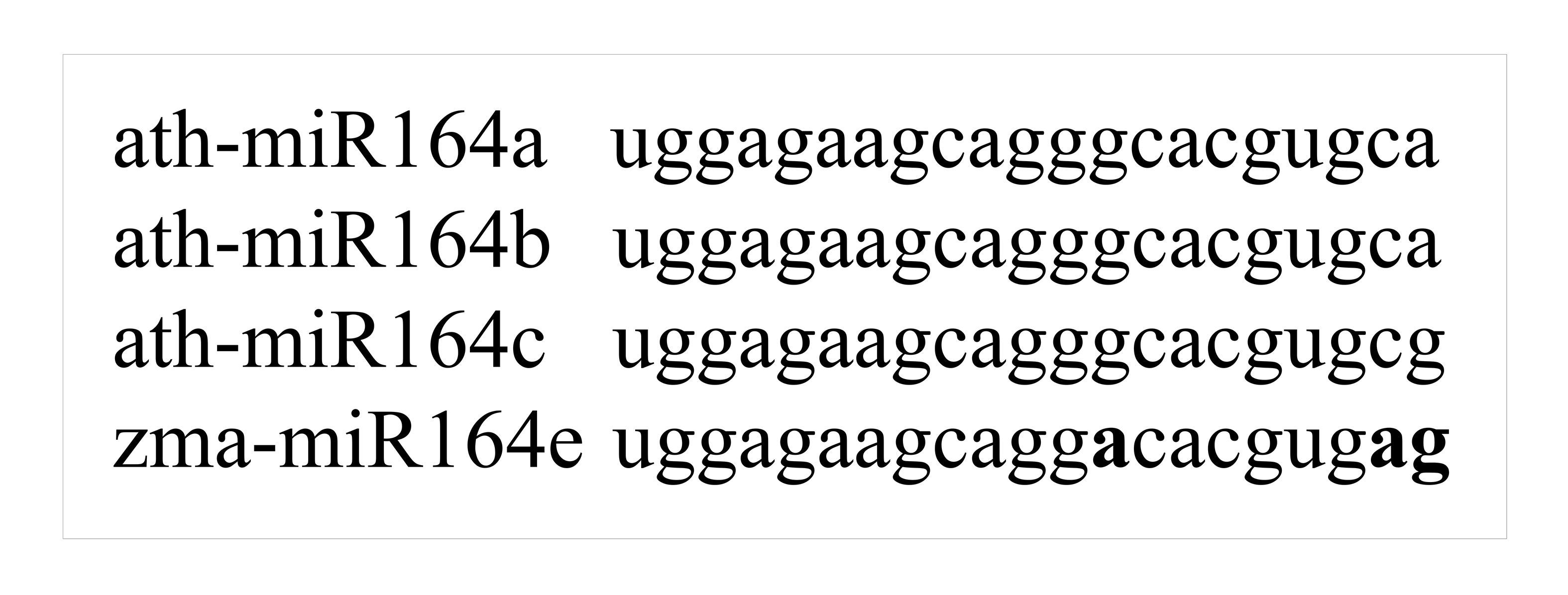

Supplement: Supplementary file 3 — Figure S3 The mature sequences of ath‐miR164 family members and zma‐miR164e. [file PBI-18-207-s008.tif]

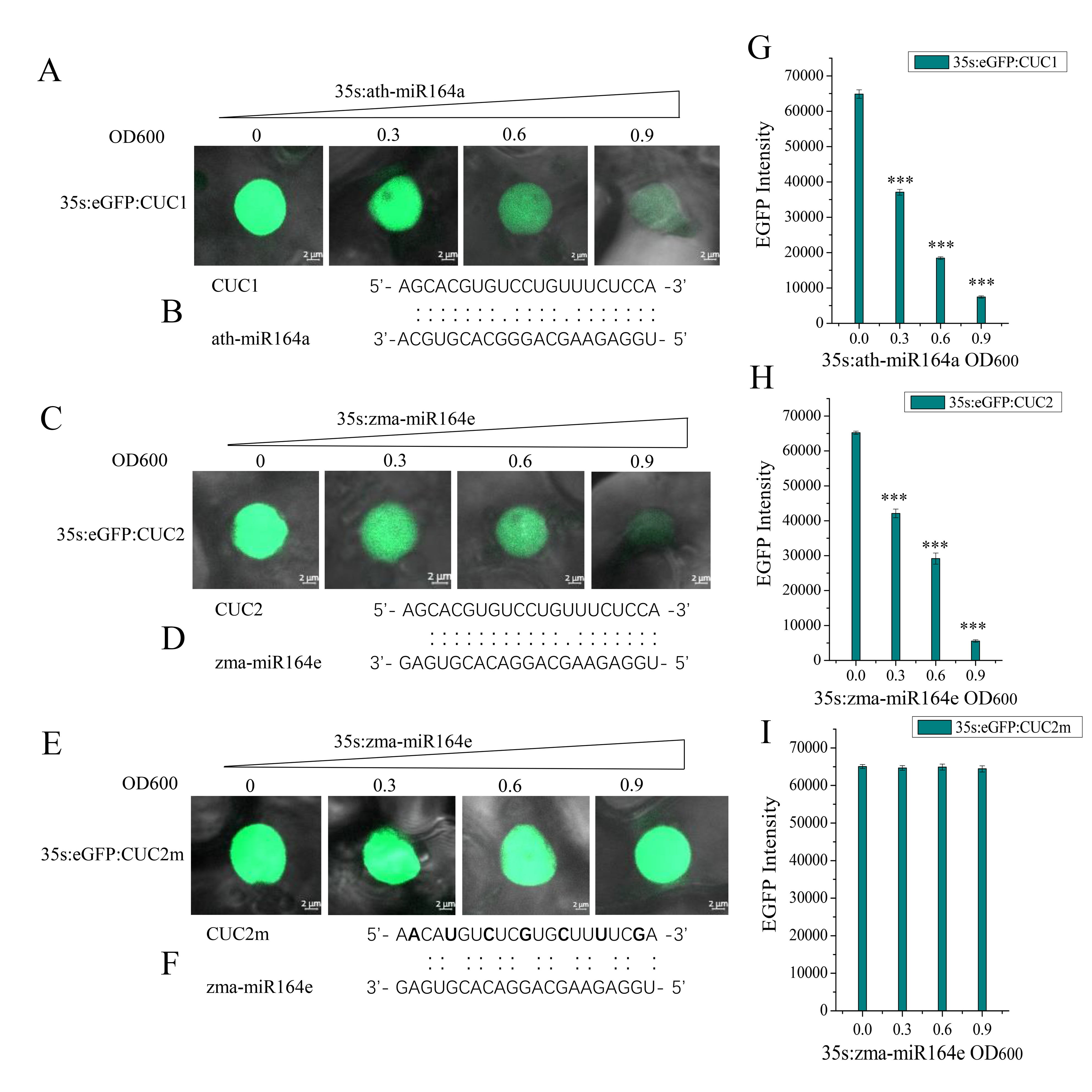

Supplement: Supplementary file 4 — Figure S4 Zma‐miR164e‐directed cleaves Arabidopsis CUC2 and decreases the accumulation of the CUC2 protein. [file PBI-18-207-s005.tif]

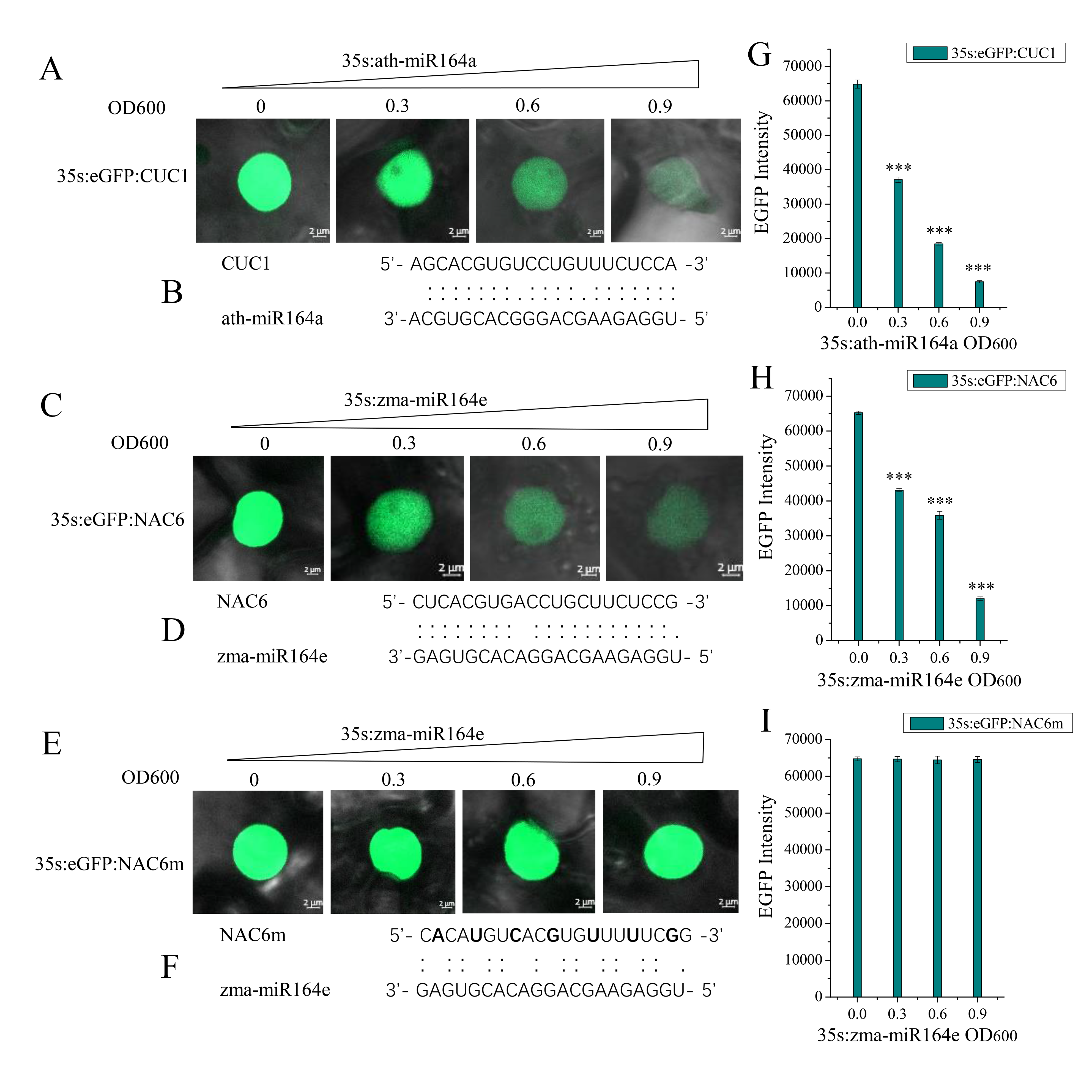

Supplement: Supplementary file 5 — Figure S5 Zma‐miR164e‐directed cleaves Arabidopsis NAC6 and decreases the accumulation of the NAC6 protein. [file PBI-18-207-s007.tif]

A

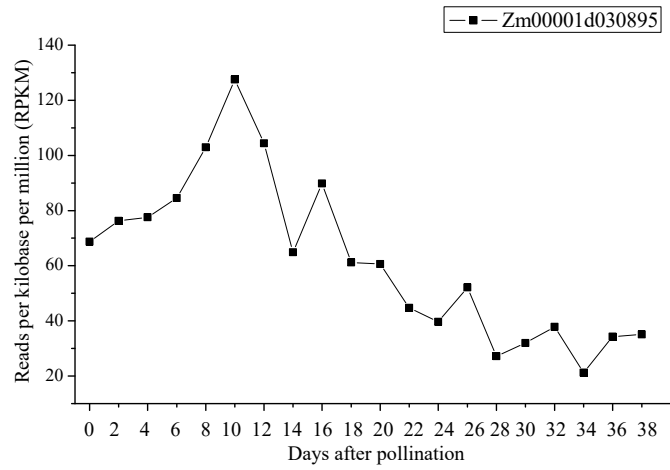

B

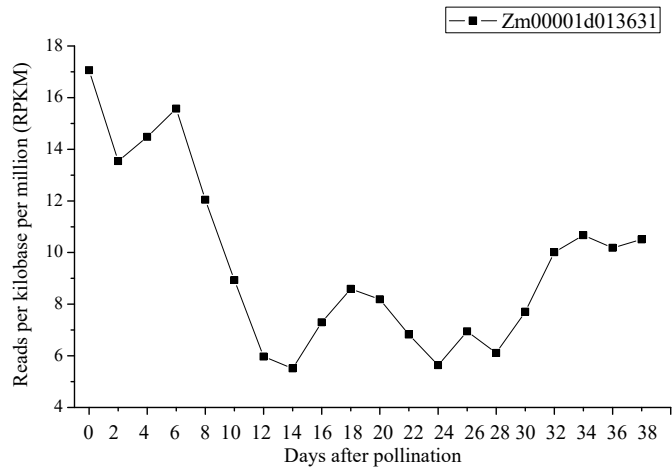

C

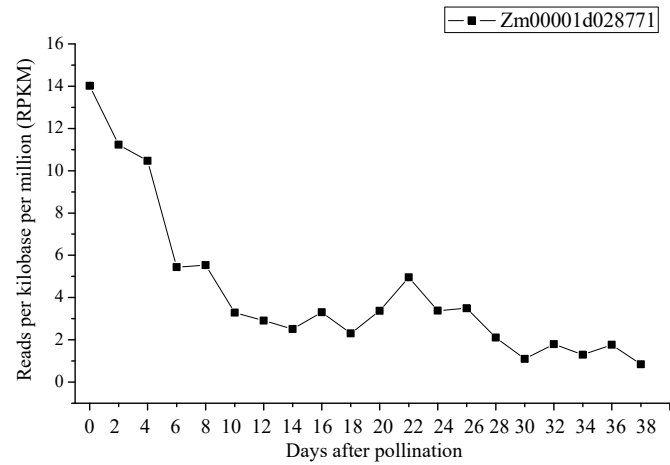

Supplement: Supplementary file 6 — Figure S6 Expression level of three genes with the top significances and stable effect in developing seed. [file PBI-18-207-s004.pdf]

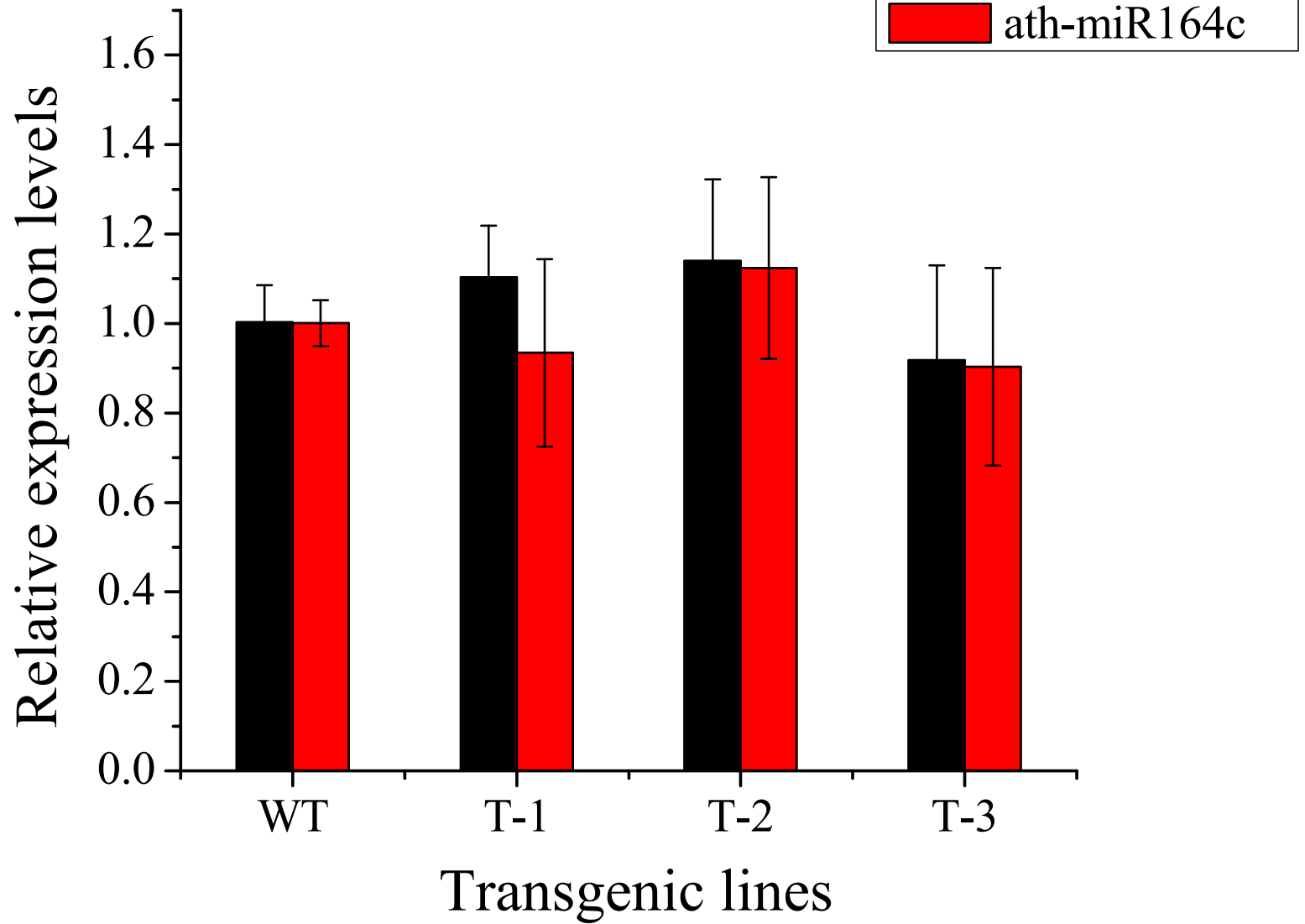

Supplement: Supplementary file 7 — Figure S7 Expression level of each member of ath‐miR164 family in the transgenic Arabidopsis plants. [file PBI-18-207-s003.pdf]
