# Supplementary material for: Analysis of the genetic architecture of maize kernel size traits by combined linkage and association mapping
Source: Plant Biotechnol J. 2019 Jun 26;18(1):207–21. doi: 10.1111/pbi.13188 (PMC6920160; doi:10.1111/pbi.13188)
Supplement: Supplementary file 8 — Table S1 Phenotypes of the 310 maize inbred lines across three environments. Table S2 Phenotypes of the IBM Syn 10 DH population across six environments. Table S3 Phenotypic correlation coefficients between the grain traits across three environments in the association panel. Table S4 Phenotypic correlation coefficients between the grain traits across six environments in the IBM Syn 10 DH population. Table S5 Environmental correlation coefficients of three grain traits in the association panel. Table S6 Environmental correlation coefficients of three grain traits in the IBM Syn 10 DH population. Table S7 Significant SNPs with stable effect for maize kernel size traits detected by GWAS using three models (GAPIT, TASSEL and FarmCPU). Table S8 Candidate genes for stable‐effect SNPs significantly associated with maize kernel size traits. Table S9 QTL identified for maize kernel size traits across seven environments using a high‐density bin map. Table S10 Co‐localized SNPs and QTL combined association and linkage mapping. Table S11 Co‐localized candidate genes by combined association and linkage mapping. Table S12 Dynamic expression patterns of the candidate genes in the transcriptomes date in developing seeds. Table S13 Candidate target genes of zma‐miR164e in Arabidopsis. Table S14 Primer sequence used in the present study. [file PBI-18-207-s002.zip › pbi13188-sup-0016-Sup-info.docx]

**Figure S1** Manhattan plots of the association analysis for KL, KW, and KT in four environments. (A) Manhattan plot of KL on ten chromosomes for the association analysis across four environments by GAPIT. The dotted red line indicates the significance threshold of *P*-value 1 × 10^−4^. The significant SNPs are labelled with red dots. ⑤, distribution of SNP markers on 10 chromosomes in association pool, the colour represents the density of the SNP markers; ①-④, represent different environments: ①, 2016 Jinghong; ②, 2016 Hongya; ③, 2016 Ya’an; ④, BLUP. Stable-effect SNPs co-detected in a multi-environment are shown in orange rectangle-shaped boxes. (B) Manhattan plot of KL on 10 chromosomes for the association analysis across four environments by TASSEL. The dotted red line indicates the significance threshold of *P*-value 1 × 10^−4^. The significant SNPs are labelled with red dots. ⑤, distribution of SNP markers on ten chromosomes in association pool, the colour represents the density of the SNP markers; ① - ④, represent different environments: ①, 2016 Jinghong; ②, 2016 Hongya; ③, 2016 Ya’an; ④, BLUP. Stable-effect SNPs co-detected in a multi-environment are shown in orange rectangle-shaped boxes. (C) Manhattan plot of KW on 10 chromosomes for the association analysis across four environments by FarmCPU. The dotted red line indicates the significance threshold of *P*-value 1 × 10^−4^. The significant SNPs are labelled with red dots. ⑤, distribution of SNP markers on ten chromosomes in association pool, the colour represents the density of the SNP markers; ① - ④, represent different environments: ①, 2016 Jinghong; ②, 2016 Hongya; ③, 2016 Ya’an; ④, BLUP. Stable-effect SNPs co-detected in a multi-environment are shown in orange rectangle-shaped boxes. (D) Manhattan plot of KW on 10 chromosomes for the association analysis across four environments by GAPIT. The dotted red line indicates the significance threshold of *P*-value 1 × 10^−4^. The significant SNPs are labelled with red dots. ⑤, distribution of SNP markers on ten chromosomes in association pool, the colour represents the density of the SNP markers; ① - ④, represent different environments: ①, 2016 Jinghong; ②, 2016 Hongya; ③, 2016 Ya’an; ④, BLUP. Stable-effect SNPs co-detected in a multi-environment are shown in orange rectangle-shaped boxes. (E) Manhattan plot of KW on 10 chromosomes for the association analysis across four environments by TASSEL. The dotted red line indicates the significance threshold of *P*-value 1 × 10^−4^. The significant SNPs are labelled with red dots. ⑤, distribution of SNP markers on 10 chromosomes in association pool, the colour represents the density of the SNP markers; ① - ④, represent different environments: ①, 2016 Jinghong; ②, 2016 Hongya; ③, 2016 Ya’an; ④, BLUP. Stable-effect SNPs co-detected in a multi-environment are shown in orange rectangle-shaped boxes. (F) Manhattan plot of KT on 10 chromosomes for the association analysis across four environments by FarmCPU. The dotted red line indicates the significance threshold of *P*-value 1 × 10^−4^. The significant SNPs are labelled with red dots. ⑤, distribution of SNP markers on ten chromosomes in association pool, the colour represents the density of the SNP markers; ① - ④, represent different environments: ①, 2016 Jinghong; ②, 2016 Hongya; ③, 2016 Ya’an; ④, BLUP. Stable-effect SNPs co-detected in a multi-environment are shown in orange rectangle-shaped boxes. (G) Manhattan plot of KT on 10 chromosomes for the association analysis across four environments by GAPIT. The dotted red line indicates the significance threshold of *P*-value 1 × 10^−4^. The significant SNPs are labelled with red dots. ⑤, distribution of SNP markers on 10 chromosomes in association pool, the colour represents the density of the SNP markers; ① - ④, represent different environments: ①, 2016 Jinghong; ②, 2016 Hongya; ③, 2016 Ya’an; ④, BLUP. Stable-effect SNPs co-detected in a multi-environment are shown in orange rectangle-shaped boxes. (H) Manhattan plot of KT on 10 chromosomes for the association analysis across four environments by TASSEL. The dotted red line indicates the significance threshold of *P*-value 1 × 10^−4^. The significant SNPs are labelled with red dots. ⑤, distribution of SNP markers on 10 chromosomes in association pool, the colour represents the density of the SNP markers; ① - ④, represent different environments: ①, 2016 Jinghong; ②, 2016 Hongya; ③, 2016 Ya’an; ④, BLUP. Stable-effect SNPs co-detected in a multi-environment are shown in orange rectangle-shaped boxes.

**Figure S2** Quantile-quantile plots for the association study of kernel size traits in maize. Quantile-quantile plots for KL, KW and KT with three association analysis models including FarmCPU, GAPIT, and TASSEL.

**Figure S3** The mature sequences of ath-miR164 family members and zma-miR164e. The sequence of zma-miR164e is different from any member of miR164 family in *Arabidopsis.*

**Figure S4** Zma-miR164e-directed cleaves *Arabidopsis CUC2* and decreases the accumulation of the *CUC2* protein. A, the eGFP:CUC1 (OD_600 nm_ = 0.6) was transiently expressed alone or co-expressed with ath-miR164e (OD_600 nm_ = 0.3/0.6/0.9) in tobacco leaf cells. The eGFP:CUC1 protein accumulation decreased with the increase of ath-miR164a concentration. The result served as positive control for this experiment. B, binding sites of ath-miR164a and *CUC1*. C, the eGFP:CUC2 (OD_600 nm_ = 0.6) was transiently expressed alone or co-expressed with zma-miR164e (OD_600 nm_ = 0.3/0.6/0.9) in tobacco leaf cells. The eGFP:CUC2 protein accumulation decreased with the increase of zma-miR164e concentration. D, binding sites of zma-miR164e and *CUC2*. E, shows that zma-miR164e cannot suppress the protein accumulation of eGFP:CUC2m whose binding sites sequence was synonymous mutated. F, synonymously mutated sequence of binding sites in *CUC2*. H and I, eGFP intensity change of eGFP:CUC1 and eGFP:CUC1m with the increase of zma-miR164e concentration, G, eGFP intensity change of eGFP:CUC1 with the increase of ath-miR164a concentration. The data for each sample were the average of the randomly detected 10 nuclei. Same results were obtained in three independent experiments. (Error bars indicate ± SD. *T*-test; ***, *P* < 0.001).

**Figure S5** Zma-miR164e-directed cleaves *Arabidopsis NAC6* and decreases the accumulation of the *NAC6* protein. A, the eGFP:CUC1 (OD_600 nm_ = 0.6) was transiently expressed alone or co-expressed with ath-miR164e (OD_600 nm_ = 0.3/0.6/0.9) in tobacco leaf cells. The eGFP:CUC1 protein accumulation decreased with the increase of ath-miR164a concentration. The result serves as a positive control for this experiment. B, binding sites of ath-miR164a and *CUC1*. C, the eGFP:NAC6 (OD_600 nm_ = 0.6) was transiently expressed alone or co-expressed with zma-miR164e (OD_600 nm_ = 0.3/0.6/0.9) in tobacco leaf cells. The eGFP:NAC6 protein accumulation decreased with the increase of zma-miR164e concentration. D, binding sites of zma-miR164e and *NAC6*. E, shows that zma-miR164e cannot suppress the protein accumulation of eGFP:NAC6m whose binding sites sequence harboured synonymous mutations. F, synonymous mutation of the binding sites in *NAC6*. H and I, eGFP intensity change of eGFP:NAC6 and eGFP:NAC6m with the increase of zma-miR164e concentration, G, eGFP intensity change of eGFP:CUC1 with increasing ath-miR164a concentrations. The data for each sample were the average of the randomly detected 10 nuclei. Same results were obtained in three independent experiments. (Error bars indicate ± SD. *T*-test; ***, *P* < 0.001).

**Figure S6** Expression level of three genes with the top significances and stable effect in developing seed. Expression level of three genes with the top significances and stable effect in developing seed. 0–38 represents days after pollination for maize seed. Reads per kilobase per million (RPKM) represents normalized transcription level using uniquely mapped reads in maize seed transcriptome data.

**Figure S7** Expression level of each member of ath-miR164 family in the transgenic *Arabidopsis* plants. qRT-PCR analysis of ath-miR164a/b and ath-miR164c in the mixed samples of inflorescence and buds of WT and the transgenic *Arabidopsis* plants. U6 was used as internal reference to calculate the relative expression of target genes using the formula 2^− (Ct target gene–Ct ß−Tubulin)^. qRT-PCR data represent the average of three biological replicates. (Error bars indicate ± SD. *T*-test; ***, *P* < 0.001; **, *P* < 0.01).

**Table S1** Phenotypes of the 310 maize inbred lines across three environments.

**Table S2** Phenotypes of the IBM Syn 10 DH population across six environments.

**Table S3** Phenotypic correlation coefficients between the grain traits across three environments in the association panel.

**Table S4** Phenotypic correlation coefficients between the grain traits across six environments in the IBM Syn 10 DH population.

**Table S5** Environmental correlation coefficients of three grain traits in the association panel.

**Table S6** Environmental correlation coefficients of three grain traits in the IBM Syn 10 DH population.

**Table S7** Significant SNPs with stable effect for maize kernel size traits detected by GWAS using three models (GAPIT, TASSEL and FarmCPU). **Table S8** Candidate genes for stable-effect SNPs significantly associated with maize kernel size traits.

**Table S9** QTL identified for maize kernel size traits across seven environments using a high-density bin map.

**Table S10** Co-localized SNPs and QTL combined association and linkage mapping.

**Table S11** Co-localized candidate genes by combined association and linkage mapping. **Table S12** Dynamic expression patterns of the candidate genes in the transcriptomes date in developing seeds.

**Table S13** Candidate target genes of zma-miR164e in *Arabidopsis*.

**Table S14** Primer sequence used in the present study.
